# Author Correction: Mammary cell gene expression atlas links epithelial cell remodeling events to breast carcinogenesis

**DOI:** 10.1038/s42003-021-02424-3

**Published:** 2021-07-15

**Authors:** Kohei Saeki, Gregory Chang, Noriko Kanaya, Xiwei Wu, Jinhui Wang, Lauren Bernal, Desiree Ha, Susan L. Neuhausen, Shiuan Chen

**Affiliations:** 1 Department of Cancer Biology, Beckman Research Institute of City of Hope, Duarte, CA USA; 2 Integrative Genomics Core, Beckman Research Institute of City of Hope, Duarte, CA USA; 3 Department of Population Sciences, Beckman Research Institute of City of Hope, Duarte, CA USA

**Keywords:** Differentiation, Data integration, Breast cancer

Correction to: *Communications Biology* 10.1038/s42003-021-02201-2, published online 2 June 2021

The original version of the Article contained the following sentence in the Discussion regarding reference 47: “Nevertheless, a recent scATACseq study reported the presence of bipotent LP cells in adult mammary glands^47^.”

This has been updated to more accurately reflect the content of reference 47. The new text is: “The results of a recent scATACseq study support this interpretation but also suggest potential plasticity within the mammary gland^47^.”

The text has been updated in the HTML and PDF versions of the Article.
