# Supplementary material for: Mammary cell gene expression atlas links epithelial cell remodeling events to breast carcinogenesis
Source: Commun Biol. 2021 Jun 2;4:660. doi: 10.1038/s42003-021-02201-2 (PMC8172904; doi:10.1038/s42003-021-02201-2)
Supplement: Supplementary file 2 — Supplementary Information [file 42003_2021_2201_MOESM2_ESM.pdf]

Mammary cell gene expression atlas links epithelial cell remodeling events to breast carcinogenesis

Kohei Saeki[1], Gregory Chang[1] , Noriko Kanaya[1] , Xiwei Wu[2], Jinhui Wang[2], Lauren Bernal[1], Desiree Ha[1], Susan L Neuhausen[3], Shiuan Chen[*1]

[1]Department of Cancer Biology, Beckman Research Institute of City of Hope, Duarte, CA, USA,

[2]Integrative Genomics Core, Beckman Research Institute of City of Hope, Duarte, CA, USA,

[3]Department of Population Sciences, Beckman Research Institute of City of Hope, Duarte, CA, USA

Kohei Saeki *et al.*

Correspondence: Shiuan Chen, Ph.D.

Department of Cancer Biology

Beckman Research Institute of the City of Hope

1500 East Duarte Road, Duarte, CA, 91010

Tel: (626) 301-4673, Fax: (626) 301-8972

E-mail: schen@coh.org

Supplementary Table 1 Software and packages.

	Category	Version	Description	Availability
Anaconda[1]	Distribution	4.7.10	Package management	https://www.anaconda.com/
Bioconductor[2]	Distribution	3.9	Package management	https://www.bioconductor.org/
biomaRt[3]	Package	2.40.4	Data retrieval	https://bioconductor.org/packages/biomaRt/
clusterProfiler[4]	Package	3.16.1	Enrichment analysis	https://bioconductor.org/packages/release/bioc/html/clusterProfiler.html
cowplot[5]	Package	1.0.0	Visualization	https://CRAN.R-project.org/package=cowplot
CytoTRACE[6]	Package	0.1.0	Trajectory reconstruction	https://cytotrace.stanford.edu/
DoubletFinder[7]	Package	2.0.2	Multiplet inference	https://github.com/chris-mcginnis-ucsf/DoubletFinder
dplyr[8]	Package	0.8.3	Data manipulation	https://CRAN.R-project.org/package=dplyr
FactoMineR[9]	Package	1.42	PCA	https://CRAN.R-project.org/package=FactoMineR

factoextra[10]	Package	1.0.5	Visualization	https://CRAN.R-project.org/package=factoextra
genefu[11]	Package	2.16.0	Molecular subtyping	https://www.bioconductor.org/packages/release/bioc/html/genefu.html
ggcorrplot[12]	Package	0.1.3	Visualization	https://cran.r-project.org/package=ggcorrplot
ggplot2[13]	Package	3.2.1	Visualization	https://CRAN.R-project.org/package=ggplot2
ggrepel[14]	Package	0.8.1	Visualization	https://CRAN.R-project.org/package=ggrepel
ggpubr[15]	Package	0.2.3	Visualization, Statistics	https://CRAN.R-project.org/package=ggpubr
ggtern[16]	Package	3.1.0	Visualization	https://CRAN.R-project.org/package=ggtern
GSVA[17]	Package	1.32.0	GSVA	https://bioconductor.org/packages/release/bioc/html/GSVA.html
JupyterLab[18]	Environment	1.0.2	Environment	https://jupyter.org/index.html
harmony[19]	Package	1.0	scRNAseq integration	https://github.com/immunogenomics/harmony
liger[20]	Package	0.5.0	scRNAseq integration	https://github.com/welch-lab/liger

matplotlab[21]	Package	3.0.2	Visualization	https://matplotlib.org/index.html
msigdbr[22]	Package	7.0.1	Data retrieval	https://CRAN.R-project.org/package=msigdbr
NumPy[23]	Package	1.17.2	Data manipulation	https://numpy.org/
pandas[24]	Package	0.25.1	Data manipulation	https://pandas.pydata.org/pandas-docs/stable/index.html
pheatmap[25]	Package	1.0.12	Visualization	https://CRAN.R-project.org/package=pheatmap
plotly[26]	Package	4.9.0	Visualization	https://CRAN.R-project.org/package=plotly
Python[27]	Language	3.7.3	Language	https://www.python.org/
R[28]	Language	3.6.1	Language	https://www.r-project.org/
reshape2[29]	Package	1.4.3	Data manipulation	https://CRAN.R-project.org/package=reshape2
RStudio[30]	Environment	1.2.5001	Environment	https://rstudio.com/
scAlign[31]	Package	1.3.0	scRNAseq integration	https://github.com/quon-titative-biology/scAlign
Seurat[32]	Package	3.1.0	scRNAseq processing	https://CRAN.R-project.org/package=Seurat
stream[33]	Package	0.3.9	Trajectory reconstruction	https://github.com/pinellolab/STREAM

stringr[34]	Package	1.4.0	Data manipulation	https://CRAN.R-project.org/package=stringr
SummarizedExperiment[35]	Package	1.14.1	Data manipulation	http://bioconductor.org/packages/release/bioc/html/SummarizedExperiment.html
TCGAbiolinks[36]	Package	2.1.2.6	Data retrieval	https://bioconductor.org/packages/release/bioc/html/TCGAbiolinks.html
TFEA.ChIP[37]	Package	1.4.2	Data retrieval	https://bioconductor.org/packages/release/bioc/html/TFEA.ChIP.html
umap[38]	Package	0.2.5.0	UMAP	https://CRAN.R-project.org/package=umap
viridis[39]	Package	0.5.1	Color palette	https://CRAN.R-project.org/package=viridis
wesanderson[40]	Package	0.3.6	Color palette	https://CRAN.R-project.org/package=wesanderson

Links were checked on 4-13-2021 for validity.

Supplementary Figures

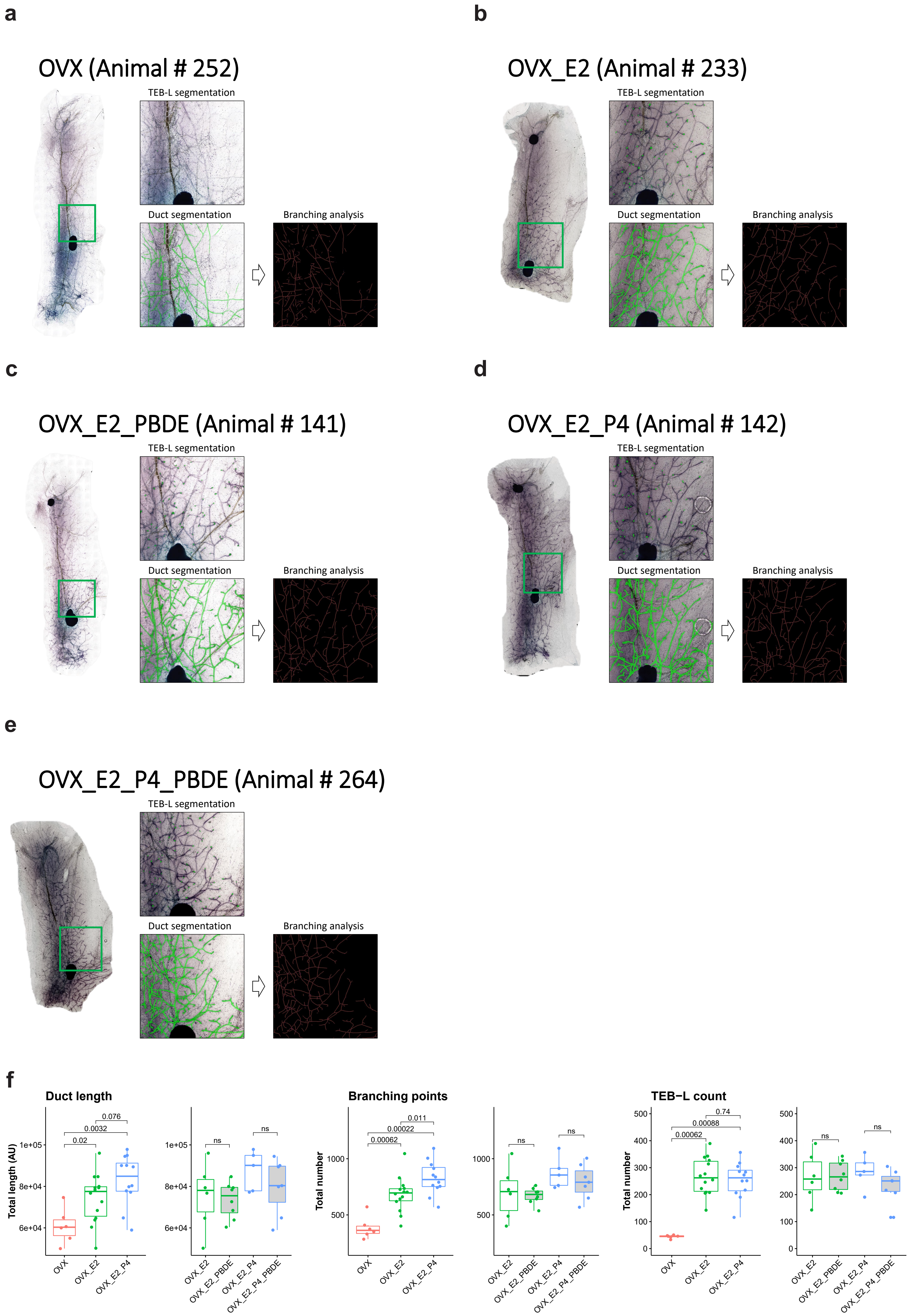

a
OVX (Animal # 252)
TEB-L segmentation
Duct segmentation
Branching analysis
b
OVX_E2 (Animal # 233)
TEB-L segmentation
Duct segmentation
Branching analysis
c
OVX_E2_PBDE (Animal # 141)
TEB-L segmentation
Duct segmentation
Branching analysis
d
OVX_E2_P4 (Animal # 142)
TEB-L segmentation
Duct segmentation
Branching analysis
e
OVX_E2_P4_PBDE (Animal # 264)
TEB-L segmentation
Duct segmentation
Branching analysis
f
Duct length
Total length (AU)
0.02
0.0032
0.076
ns
ns
Branching points
Total number
0.00062
0.00022
0.011
ns
ns
TEB−L count
Total number
0.00062
0.00088
0.74
ns
ns
OVX
OVX_E2
OVX_E2_P4
OVX_E2_PBDE
OVX_E2_P4_PBDE

Supplementary Figure 1 Reorganization of the surgically menopaused glands after the HRTs and PBDEs exposure. (**a-e**) The representative whole gland staining images from OVX (n=6) (**a**), OVX_E2 (n=6) (**b**), OVX_E2_PBDE (n=8) (**c**), OVX_E2_P4 (n=5) (**d**), and OVX_E2_P4_PBDE (n=7) groups (**e**). In each image, the entire gland, the results of the segmentation of the terminal end bud-like structures and the ductal structure, and the skeletonized image for the branching analysis are presented. (**f**) The changes by different HRTs and the addition of PBDEs on the total ductal length, total number of the branching points, and TEB-L counts were summarized and compared between the groups. The n number is described above (biologically independent samples). Cliff's delta values for the comaprisons of duct length between OVX and OVX_E2, OVX and OVX_E2_P4, and OVX_E2 and OVX_E2_P4 were 0.67 (CI: 0.17-0.89), 0.83 (CI: 0.39-0.96), and 0.42 (0.07-0.74), respectively. Cliff's delta values for the comaprisons of branching points between OVX and OVX_E2, OVX and OVX_E2_P4, and OVX_E2 and OVX_E2_P4 were 0.90 (CI: 0.59-0.98), 0.97 (CI: 0.83-0.99), and 0.58 (CI: 0.11-0.84), respectively. Cliff's delta values for the comaprisons of TEB-L counts between OVX and OVX_E2, OVX and OVX_E2_P4 were 1.00 (CI: 0.96-1.00) and 1.00 (CI: 0.96-1.00), respectively. ns; not significant. CI; confidence interval. The box-plot elements were defined as follows: center line, median; box limits, upper and lower quartiles; whiskers, 1.5x interquartile range; points, outliers.

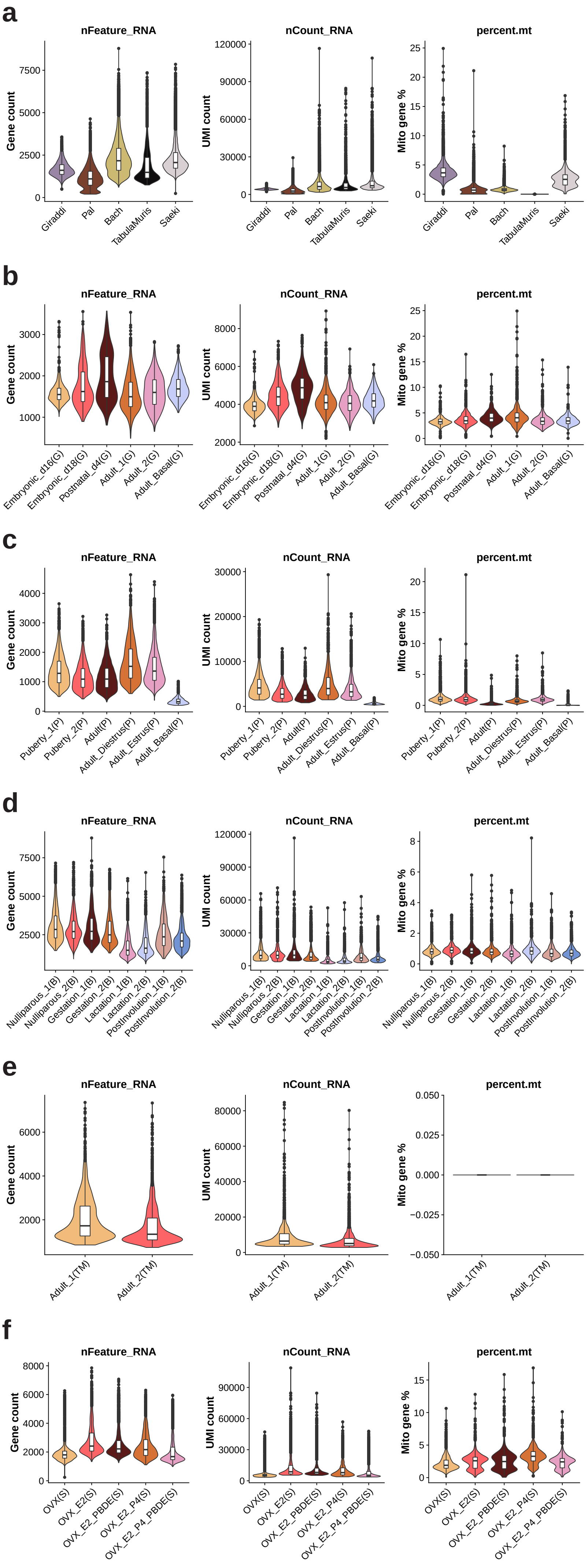
a
nFeature_RNA
Gene count
Giraddi
Pal
Bach
TabulaMuris
Saeki
nCount_RNA
UMI count
percent.mt
Mito gene %
b
Embryonic_d16(G)
Embryonic_d18(G)
Postnatal_d4(G)
Adult_1(G)
Adult_2(G)
Adult_Basal(G)
c
Puberty_1(P)
Puberty_2(P)
Adult(P)
Adult_Diestrus(P)
Adult_Estrus(P)
Adult_Basal(P)
d
Nulliparous_1(B)
Nulliparous_2(B)
Gestation_1(B)
Gestation_2(B)
Lactation_1(B)
Lactation_2(B)
PostInvolution_1(B)
PostInvolution_2(B)
e
Adult_1(TM)
Adult_2(TM)
f
OVX(S)
OVX_E2(S)
OVX_E2_PBDE(S)
OVX_E2_P4(S)
OVX_E2_P4_PBDE(S)

Supplementary Figure 2 Qualitative characteristics of each dataset and each biological sample. (**a-f**) Distribution of the number of detected genes (nFeature_RNA) and transcripts (nCount_RNA) and the percentage of the mitochondrial genes (percent.mt) was compared between the five datasets (**a**) and within the Giraddi et al. dataset (**b**), the Pal et al. dataset (**c**), the Bach et al. dataset (**d**), the TabulaMuris dataset (**e**), and this dataset (**f**). The n number of each study and sample is summarized in Supplementary Data 2. The box-plot elements were defined as follows: center line, median; box limits, upper and lower quartiles; whiskers, 1.5x interquartile range; points, outliers. UMI; unique molecular identifier.

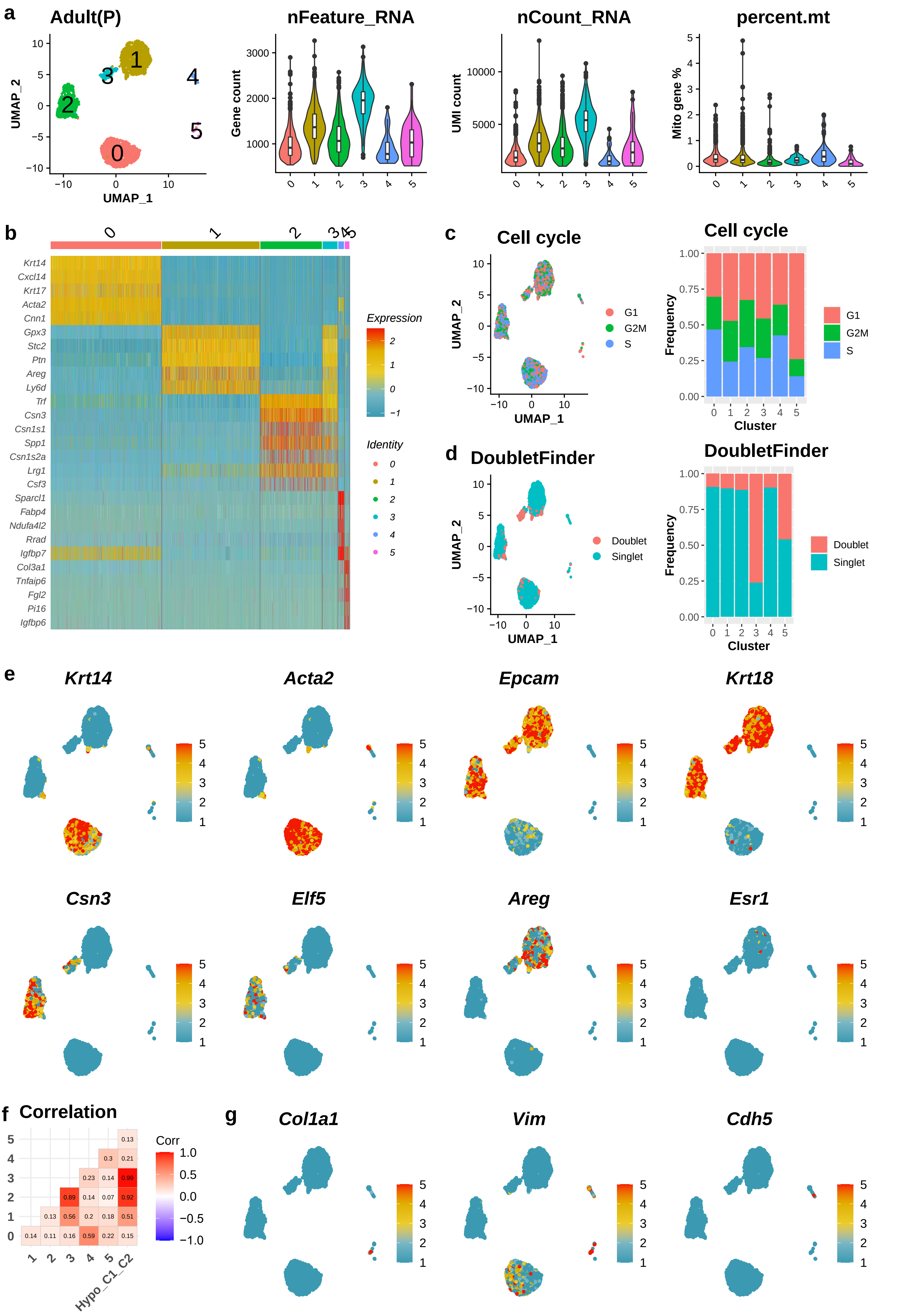
a
Adult(P)
UMAP_2
UMAP_1
nFeature_RNA
Gene count
nCount_RNA
UMI count
percent.mt
Mito gene %
b
Krt14
Cxcl14
Krt17
Acta2
Cnn1
Gpx3
Stc2
Ptn
Areg
Ly6d
Trf
Csn3
Csn1s1
Spp1
Csn1s2a
Lrg1
Csf3
Sparcl1
Fabp4
Ndufa4l2
Rrad
Igfbp7
Col3a1
Tnfaip6
Fgl2
Pi16
Igfbp6
Expression
Identity
c
Cell cycle
G1
G2M
S
Frequency
Cluster
d
DoubletFinder
Doublet
Singlet
e
Krt14
Acta2
Epcam
Krt18
Csn3
Elf5
Areg
Esr1
f
Correlation
Corr
Hypo_C1_C2
g
Col1a1
Vim
Cdh5

Supplementary Figure 3 Preprocessing of the scRNAseq data from the adult virgin mammary gland in the Pal et al. dataset. (**a**) The results of dimension reduction and clustering on a UMAP plot and distribution of nFeature_RNA, nCount_RNA, and percent.mt values in each cluster. The n number of each cluster is summarized in Supplementary Data 2. The box-plot elements were defined as follows: center line, median; box limits, upper and lower quartiles; whiskers, 1.5x interquartile range; points, outliers. (**b**) The heatmap showing the expression of the top five genes in each cluster. (**c**) The result of the cell cycle scoring was projected on a UMAP plot and summarized in a stacked bar plot. (**d**) The results of the *DoubletFinder* analysis was projected on a UMAP plot and summarized in a stacked bar plot. (**e**) The expression of the marker genes for the mammary epithelium were expressed on UMAP plots (*Krt14* and *Acta2*; Basal cells, *Epcam* and *Krt18*; Luminal cells, *Csn3* and *Elf5*; Luminal alveolar cells, and *Areg* and *Esr1*; Luminal hormone-sensing cells). (**f**) The results of the correlation analysis of pseudo bulk RNAseq analysis, including a hypothetical doublet cluster between C1 and C2 (Hypo_C1_C2). (**g**) The expression of marker genes for contaminating stromal cells were visualized on UMAP plots (*Col1a1*; Fibroblasts, *Vim*; Mesenchymal cells, *Cdh5*; Endothelial cells). UMI; unique molecular identifier.

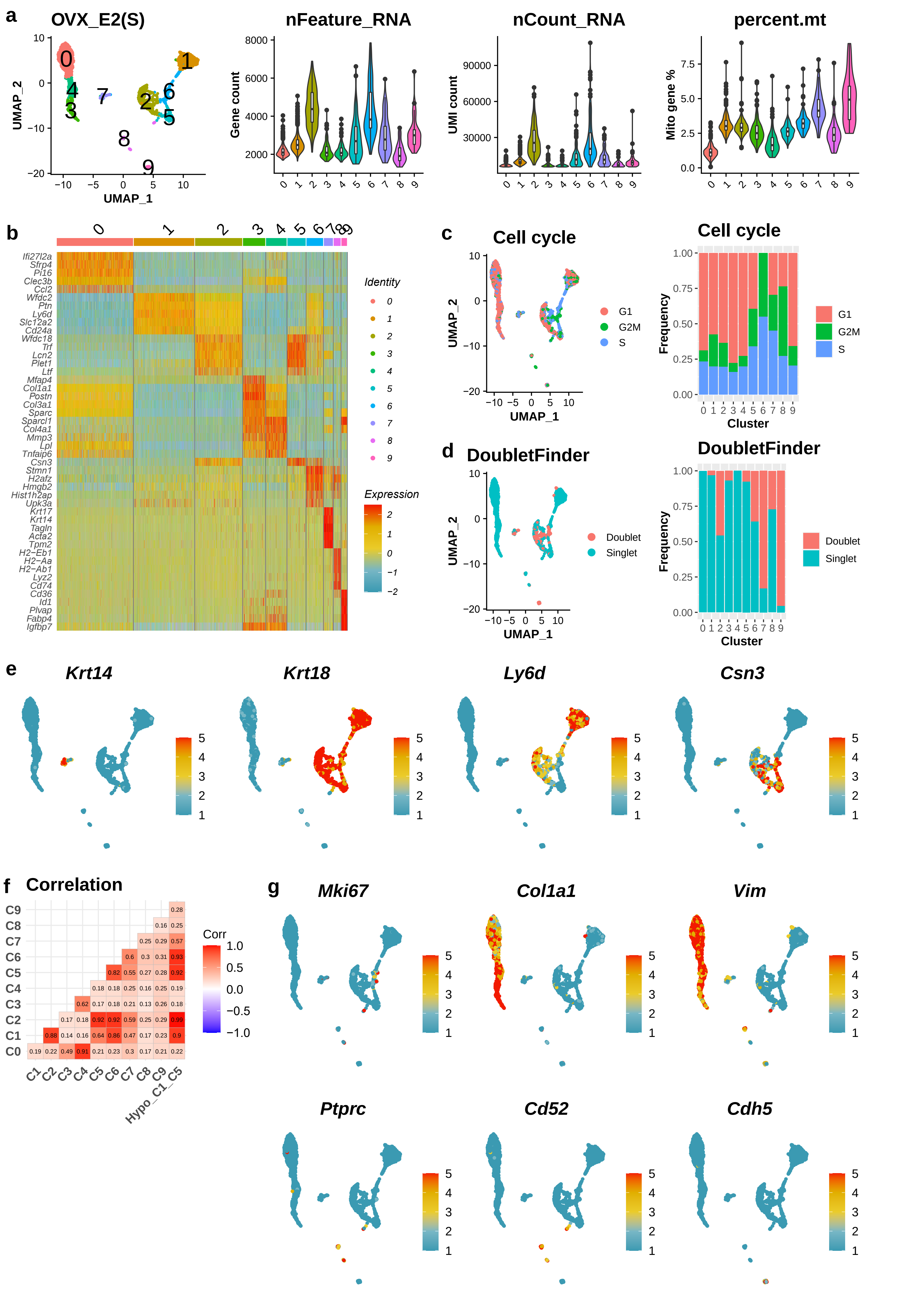

a
OVX_E2(S)
nFeature_RNA
nCount_RNA
percent.mt
UMAP_1
UMAP_2
Gene count
UMI count
Mito gene %
b
Identity
Expression
c
Cell cycle
G1
G2M
S
Frequency
Cluster
d
DoubletFinder
Doublet
Singlet
e
Krt14
Krt18
Ly6d
Csn3
f
Correlation
Corr
g
Mki67
Col1a1
Vim
Ptprc
Cd52
Cdh5

Supplementary Figure 4 Preprocessing of the scRNAseq data of the mammary gland from the surgically menopaused mouse treated with estrogen for a week in this study. (**a**) The results of dimension reduction and clustering on a UMAP plot and distribution of nFeature_RNA, nCount_RNA, and percent.mt values in each cluster. The n number of each cluster is summarized in Supplementary Data 2. The box-plot elements were defined as follows: center line, median; box limits, upper and lower quartiles; whiskers, 1.5x interquartile range; points, outliers. (**b)** The heatmap showing the expression of the top five genes in each cluster. (**c**) The results of cell cycle scoring were projected on a UMAP plot and summarized in a stacked bar plot. (**d**) The results of the *DoubletFinder* analysis was projected on a UMAP plot and summarized in a stacked bar plot. (**e**) The expression of the marker genes for the mammary epithelium was expressed on UMAP plots (*Krt14*; Basal cells, *Krt18*; Luminal cells, *Csn3*; Luminal alveolar cells, and *Ly6d*; Luminal hormone-sensing cells). (**f**) The results of the correlation analysis of pseudo bulk RNAseq analysis, including a hypothetical doublet cluster between C1 and C5 (Hypo_C1_C5). (**g**) The expression of marker genes for proliferating cells and contaminating stromal cells were visualized on UMAP plots (*Mki67*; Proliferating cells, *Col1a1*; Fibroblasts, *Vim*; Mesenchymal cells, *Ptprc*; Hematopoietic cells, *Cd52*; Macrophages, *Cdh5*; Endothelial cells). UMI; unique molecular identifier.

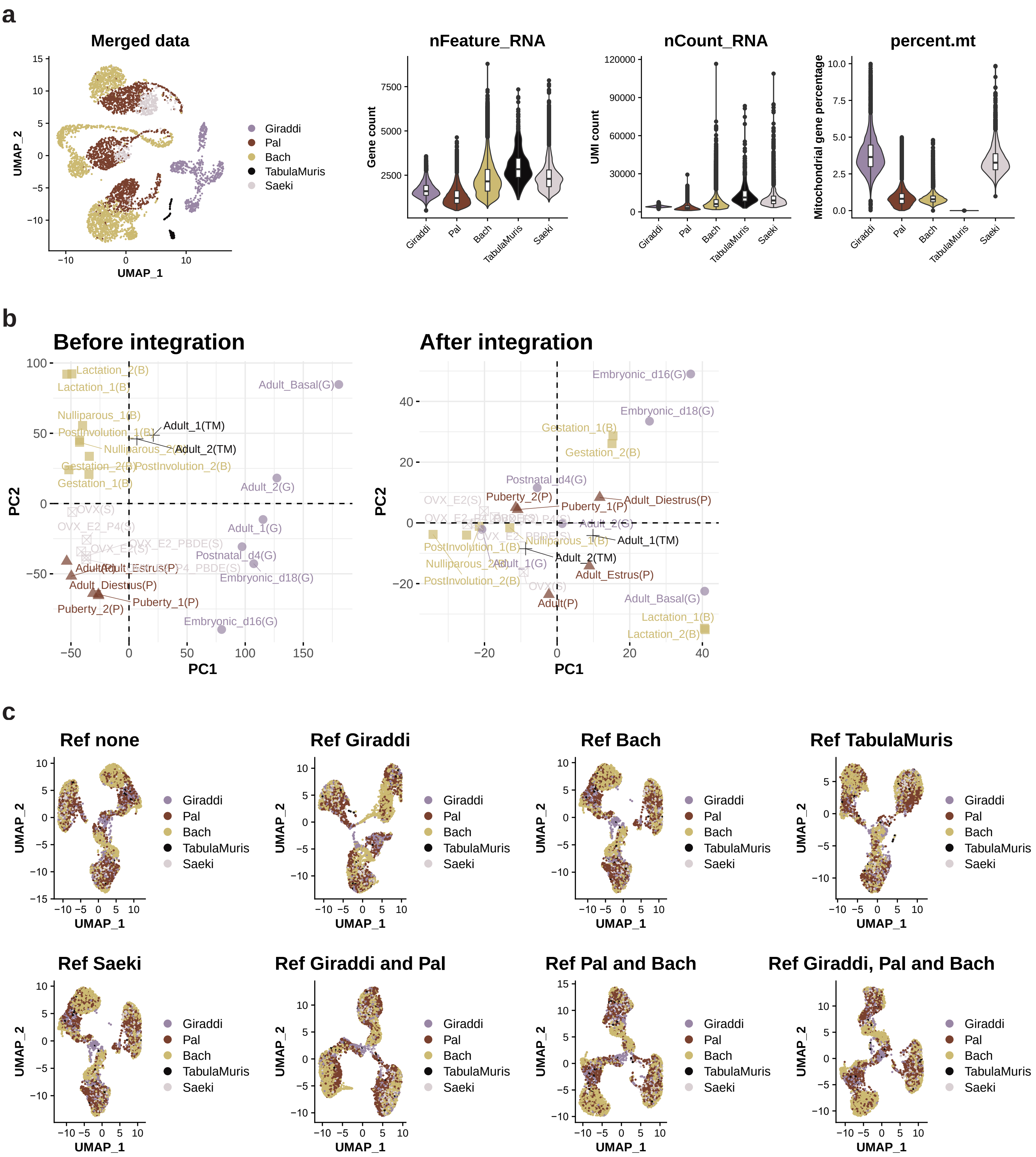

a
Merged data
UMAP_2
UMAP_1
Giraddi
Pal
Bach
TabulaMuris
Saeki
nFeature_RNA
Gene count
nCount_RNA
UMI count
percent.mt
Mitochondrial gene percentage
b
Before integration
After integration
PC1
PC2
Lactation_2(B)
Lactation_1(B)
Adult_Basal(G)
Nulliparous_1(B)
Postinvolution_1(B)
Adult_1(TM)
Adult_2(TM)
Postinvolution_2(B)
Gestation_1(B)
Gestation_2(B)
Adult_2(G)
Adult_1(G)
OVX(S)
Postnatal_d4(G)
Embryonic_d18(G)
Embryonic_d16(G)
Adult_Diestrus(P)
Adult_Estrus(P)
Puberty_1(P)
Puberty_2(P)
Adult(P)
c
Ref none
Ref Giraddi
Ref Bach
Ref TabulaMuris
Ref Saeki
Ref Giraddi and Pal
Ref Pal and Bach
Ref Giraddi, Pal and Bach

Supplementary Figure 5 Data integration of the five scRNAseq datasets. (**a**) Visualization of the preprocessed and merged datasets on a UMAP plot and distribution of nFeature_RNA, nCount_RNA, and percent.mt in each preprocessed dataset. The n number of each study after preprocessing is summarized in Supplementary Data 2. The box-plot elements were defined as follows: center line, median; box limits, upper and lower quartiles; whiskers, 1.5x interquartile range; points, outliers. Five thousands cells were sampled from the entire data for UMAP visualization. (**b**) The results of pseudo bulk RNAseq of each sample were color-coded by datasets before and after the anchor-based data integration by *Seurat v3*. (**c**) Visualization of the data in UMAP dimensionality after the anchor-based data integration using the different dataset(s) as references. Ref none; the datasets were integrated without any reference. Five thousands cells were sampled from the entire data for UMAP visualization. UMI; unique molecular identifier.

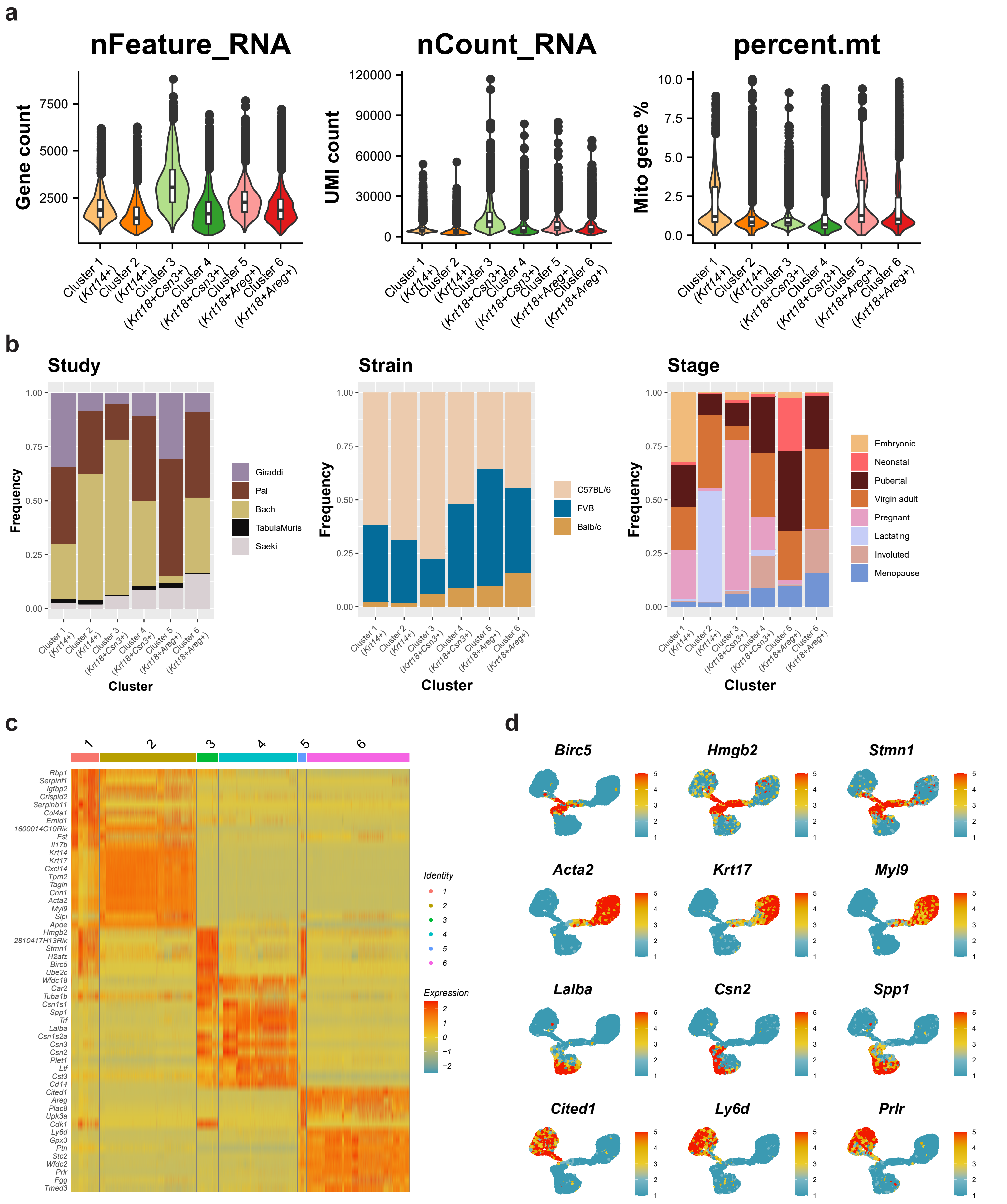
a
nFeature_RNA
nCount_RNA
percent.mt
Gene count
UMI count
Mito gene %
7500
5000
2500
120000
90000
60000
30000
0
10.0
7.5
5.0
2.5
0.0
Cluster 1 (Krt14+)
Cluster 2 (Krt14+)
Cluster 3 (Krt18+Csn3+)
Cluster 4 (Krt18+Csn3+)
Cluster 5 (Krt18+Areg+)
Cluster 6 (Krt18+Areg+)
b
Study
Strain
Stage
Frequency
Cluster
1.00
0.75
0.50
0.25
0.00
Giraddi
Pal
Bach
TabulaMuris
Saeki
C57BL/6
FVB
Balb/c
Embryonic
Neonatal
Pubertal
Virgin adult
Pregnant
Lactating
Involuted
Menopause
c
1
2
3
4
5
6
Rbp1
Serpinf1
Igfbp2
Crispld2
Serpinb11
Col4a1
Emid1
1600014C10Rik
Fst
Il17b
Krt14
Krt17
Cxcl14
Tpm2
Tagln
Cnn1
Acta2
Myl9
Slpi
Apoe
Hmgb2
2810417H13Rik
Stmn1
H2afz
Birc5
Ube2c
Wfdc18
Car2
Tuba1b
Csn1s1
Spp1
Trf
Lalba
Csn1s2a
Csn3
Csn2
Plet1
Ltf
Cst3
Cd14
Cited1
Areg
Plac8
Upk3a
Cdk1
Ly6d
Gpx3
Ptn
Stc2
Wfdc2
Prlr
Fgg
Tmed3
Identity
Expression
2
1
0
−1
−2
d
Birc5
Hmgb2
Stmn1
Acta2
Krt17
Myl9
Lalba
Csn2
Spp1
Cited1
Ly6d
Prlr

Supplementary Figure 6 Features of the identified clusters in the integrated data. (**a**) Distribution of nFeature_RNA, nCount_RNA, and percent.mt values in each cluster. The n number of each cluster is as follows: C1 (n=4,217), C2 (n=14,493), C3 (n=3,176), C4 (n=11,856), C5 (n=1,156), and C6 (n=15,509) (biologically independent samples). The box-plot elements were defined as follows: center line, median; box limits, upper and lower quartiles; whiskers, 1.5x interquartile range; points, outliers. (**b**) Breakdown of each cluster by datasets (Study), mouse strains (Strain), and different developmental stages (Stage). (**c**) The heatmap showing the expression of the top five genes in each cluster. (**d**) The expression of selected genes characteristic to the putative progenitor clusters (C1, C3, and C5; *Birc5*, *Hmgb2*, *Stmn1*), the basal cell lineage (C1 and C2; *Acta2*, *Krt17*, and *Myl9*), the luminal alveolar lineage (C3 and C4; *Lalba*, *Csn2*, and *Spp1*), and the luminal hormone-sensing cells (C5 and C6; *Cited1*, *Ly6d*, and *Prlr*). Five thousands cells were sampled from the entire data for UMAP visualization. UMI; unique molecular identifier.

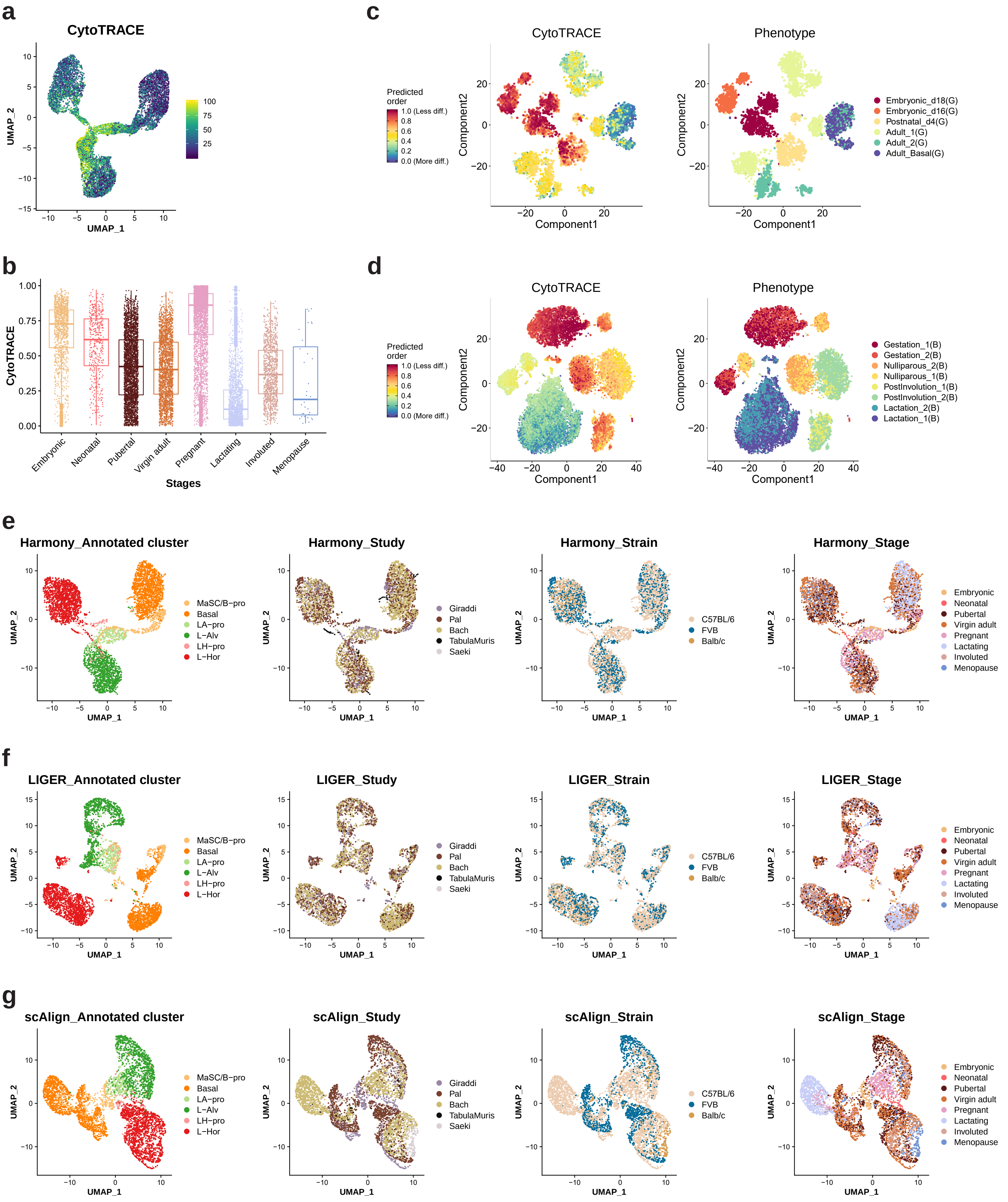

a
CytoTRACE
UMAP_2
UMAP_1
100
75
50
25
b
CytoTRACE
Embryonic
Neonatal
Pubertal
Virgin adult
Pregnant
Lactating
Involuted
Menopause
Stages
c
CytoTRACE
Phenotype
Predicted order
1.0 (Less diff.)
0.0 (More diff.)
Component1
Component2
Embryonic_d18(G)
Embryonic_d16(G)
Postnatal_d4(G)
Adult_1(G)
Adult_2(G)
Adult_Basal(G)
d
CytoTRACE
Phenotype
Gestation_1(B)
Gestation_2(B)
Nulliparous_2(B)
Nulliparous_1(B)
PostInvolution_1(B)
PostInvolution_2(B)
Lactation_2(B)
Lactation_1(B)
e
Harmony_Annotated cluster
Harmony_Study
Harmony_Strain
Harmony_Stage
MaSC/B−pro
Basal
LA−pro
L−Alv
LH−pro
L−Hor
Giraddi
Pal
Bach
TabulaMuris
Saeki
C57BL/6
FVB
Balb/c
f
LIGER_Annotated cluster
LIGER_Study
LIGER_Strain
LIGER_Stage
g
scAlign_Annotated cluster
scAlign_Study
scAlign_Strain
scAlign_Stage

Supplementary Figure 7 The results from the CytoTRACE analysis and the different integration algorithms. (**a**) Projection of the results from the Scanorama-based implementation of CytoTRACE on a UMAP plot of the integrated data by *Seurat*. A higher score indicates less differentiated states. (**b**) The CytoTRACE scores of cells in different stages. The n number of each stage is as follows: Embryonic (n=1,131), Neonatal (n=448), Pubertal (n=3,139), Virgin adult (n=2,139), Pregnant (n=3,375), Lactating (n=1,630), Involuted (n=1,068), and Menopause (n=32) (biologically independent samples). The box-plot elements were defined as follows: center line, median; box limits, upper and lower quartiles; whiskers, 1.5x interquartile range; points, outliers. (**c**) The results of the CytoTRACE analysis of the Giraddi et al. dataset. The t-Distributed Stochastic Neighbor Embedding (t-SNE) plots of the data were color-coded by the CytoTRACE score and the individual samples. (**d**) The results of the CytoTRACE analysis of the Bach et al. dataset. The t-SNE plots of the data were color-coded by the CytoTRACE score and the individual samples. (**e-g**) Data integration by additional algorithms. The UMAP plots after integration were color-coded by their original clustering in *Seurat v3*, datasets (Study), mouse strains (Strain), and different developmental stages (Stage). Five thousands cells were sampled from the entire data for UMAP visualization. (**e**) The data integrated by *Harmony*. (**f**) The data integrated by *LIGER*. (**g**) The data integrated by *scAlign*.

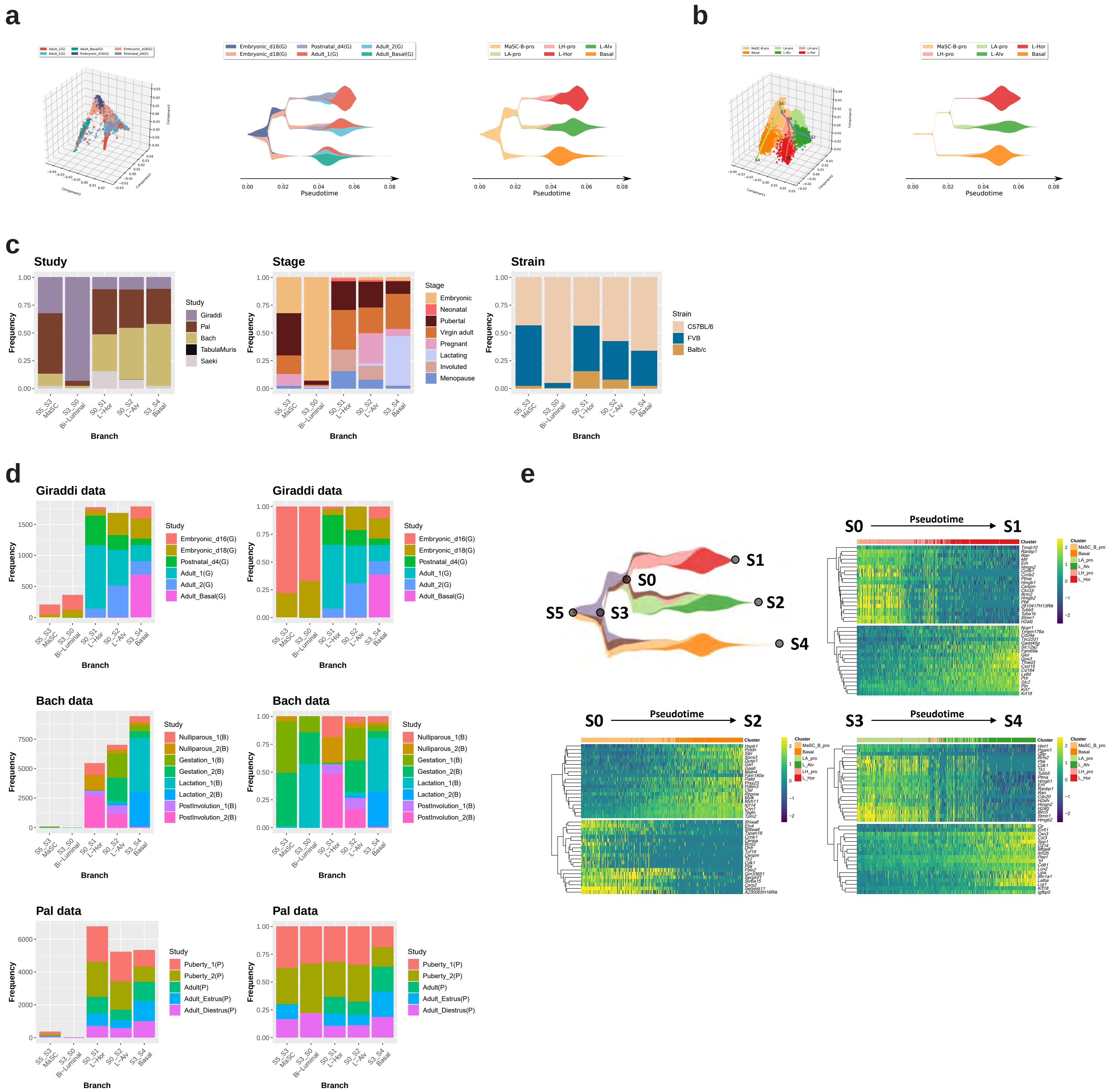

a
b
Pseudotime
c
Study
Stage
Strain
Frequency
Branch
d
Giraddi data
Bach data
Pal data
e
S0
S1
S2
S3
S4
S5
Pseudotime

Supplementary Figure 8 STREAM analysis. (**a**) The base trajectory was built using the Giraddi et al. dataset, projected in Modified Locally Linear Embedding (Mlle) spaces, and plotted on stream plots, color-coded by individual samples and clusters. **(b**) The 50K mouse mammary epithelial cells were mapped onto the base trajectory, projected in the Mlle space, and plotted on a stream plot. (**c**) Breakdown of each branch by datasets (Study), mouse strains (Strain), and different developmental stages (Stage). (**d**) Breakdowns of Giraddi et al., Bach et al., and Pal et al. datasets shown in stacked bar plots. The absolute number and proportion of cells in each branch were presented and color-coded by samples. (**e**) Expression of the top 20 transitionally expressed genes in S0_S1 (L-Hor), S0_S2 (L-Alv), and S3_S4 (Basal) branches were shown in heatmaps. Cells were sorted in the order of the relevant psudotime.

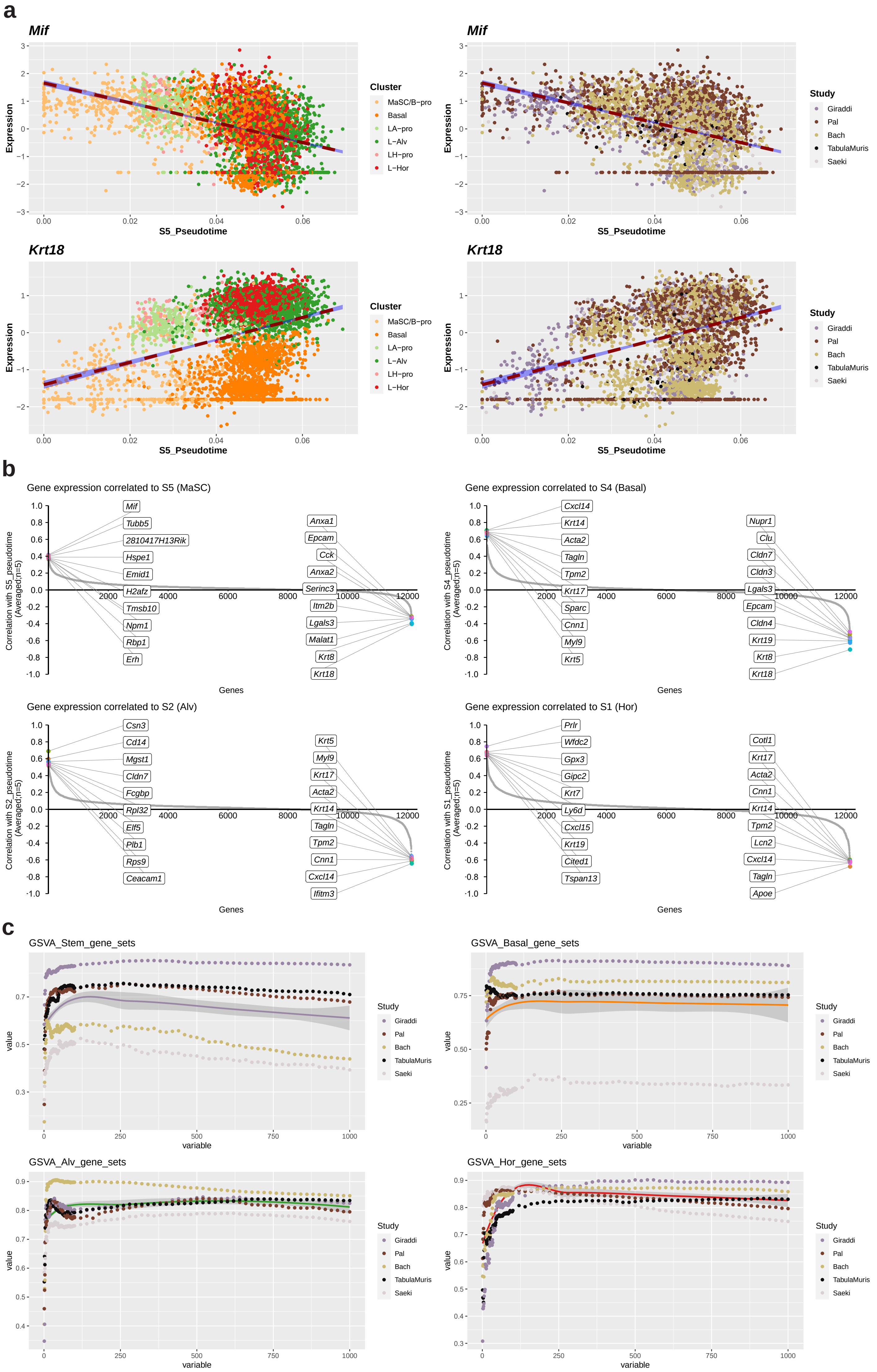
a
Mif
Expression
S5_Pseudotime
Cluster
MaSC/B−pro
Basal
LA−pro
L−Alv
LH−pro
L−Hor
Study
Giraddi
Pal
Bach
TabulaMuris
Saeki
Krt18
b
Gene expression correlated to S5 (MaSC)
Correlation with S5_pseudotime (Averaged;n=5)
Genes
Mif
Tubb5
2810417H13Rik
Hspe1
Emid1
H2afz
Tmsb10
Npm1
Rbp1
Erh
Anxa1
Epcam
Cck
Anxa2
Serinc3
Itm2b
Lgals3
Malat1
Krt8
Krt18
Gene expression correlated to S4 (Basal)
Correlation with S4_pseudotime (Averaged;n=5)
Cxcl14
Krt14
Acta2
Tagln
Tpm2
Krt17
Sparc
Cnn1
Myl9
Krt5
Nupr1
Clu
Cldn7
Cldn3
Lgals3
Epcam
Cldn4
Krt19
Krt8
Krt18
Gene expression correlated to S2 (Alv)
Correlation with S2_pseudotime (Averaged;n=5)
Csn3
Cd14
Mgst1
Cldn7
Fcgbp
Rpl32
Elf5
Plb1
Rps9
Ceacam1
Krt5
Myl9
Krt17
Acta2
Krt14
Tagln
Tpm2
Cnn1
Cxcl14
Ifitm3
Gene expression correlated to S1 (Hor)
Correlation with S1_pseudotime (Averaged;n=5)
Prlr
Wfdc2
Gpx3
Gipc2
Krt7
Ly6d
Cxcl15
Krt19
Cited1
Tspan13
Cotl1
Krt17
Acta2
Cnn1
Krt14
Tpm2
Lcn2
Cxcl14
Tagln
Apoe
c
GSVA_Stem_gene_sets
GSVA_Basal_gene_sets
GSVA_Alv_gene_sets
GSVA_Hor_gene_sets
value
variable

Supplementary Figure 9 Identification of the lineage-specific genes and gene sets. (**a**) The expression of the top positively (*Mif*) and negatively (*Krt18*) correlated genes with S5 pseudotime (“Stem” state). The X-axis represents the passage of pseudotime from the S5 node and the Y-axis represents gene expression that was individually scaled in each dataset. Each point represents a single cell. The points were color-coded by clusters and datasets. The dashed lines indicate regression lines. Five thousands cells were sampled from the entire data for UMAP visualization. (**b**) The results of the comprehensive correlation analysis between the gene expression and the pseudotimes. The combinations of the 12,319 genes and the four different pseudotimes [S5; Stem, S4; Basal, S2; Alv, and S1; Hor] were considered. The genes were ordered according to the correlation coefficients and the top 10 positively and negatively correlated genes were labeled. The X-axis represents the rank order and the Y-axis represents the averaged correlation coefficients from the five datasets. (**c**) The change in the performance of the gene sets according to the number of the top correlated genes in a gene set. The X-axis represents the number of gene(s) in a gene set and the Y-axis represents the correlation between the scGSVA score and the pseudotime [S5; Stem, S4; Basal, S2; Alv, and S1; Hor]. The correlation was calculated on a dataset basis. The solid lines represent regression curves in a LOESS model and the surrounding grey areas represent the confidence intervals.

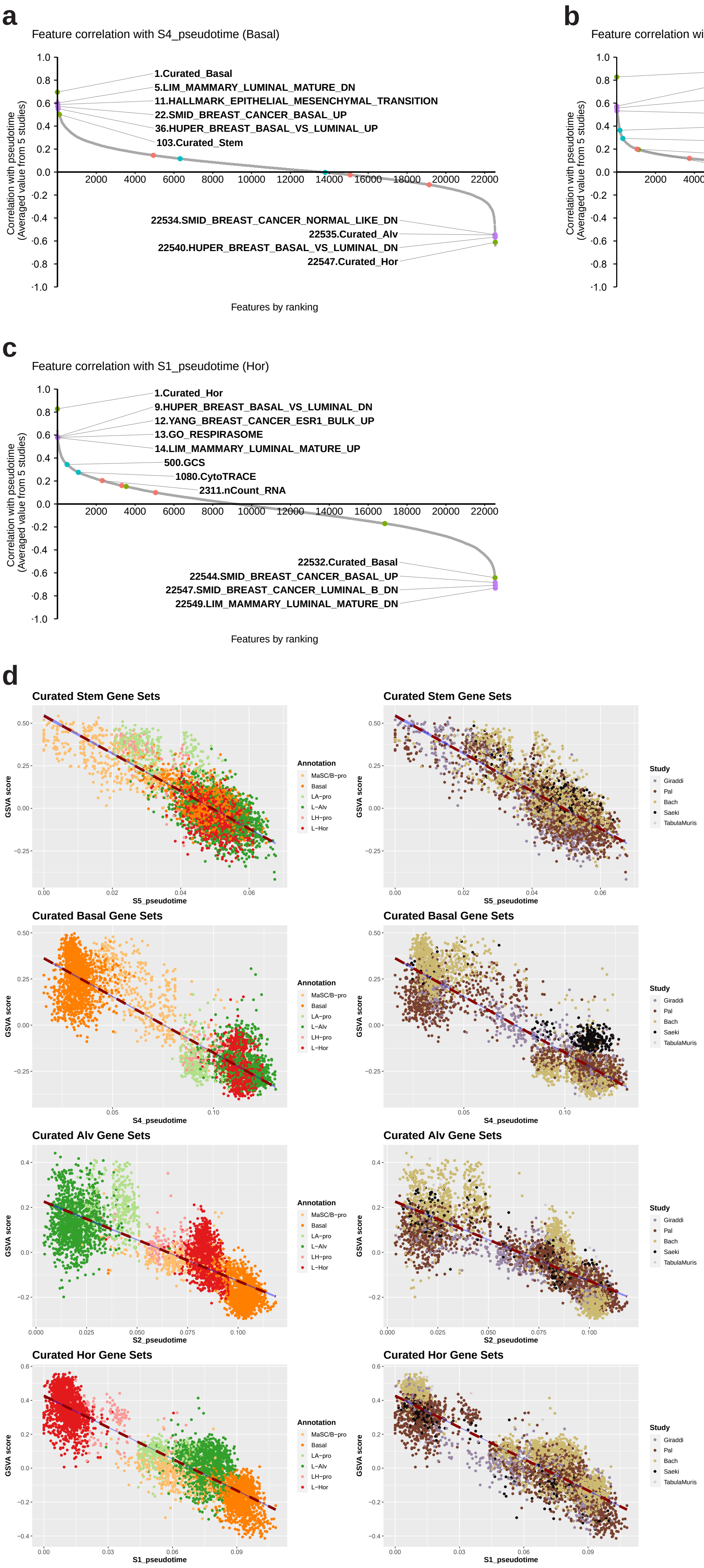

a
Feature correlation with S4_pseudotime (Basal)
Correlation with pseudotime
(Averaged value from 5 studies)
1.Curated_Basal
5.LIM_MAMMARY_LUMINAL_MATURE_DN
11.HALLMARK_EPITHELIAL_MESENCHYMAL_TRANSITION
22.SMID_BREAST_CANCER_BASAL_UP
36.HUPER_BREAST_BASAL_VS_LUMINAL_UP
103.Curated_Stem
22534.SMID_BREAST_CANCER_NORMAL_LIKE_DN
22535.Curated_Alv
22540.HUPER_BREAST_BASAL_VS_LUMINAL_DN
22547.Curated_Hor
Features by ranking
b
Feature correlation with S2_pseudotime (Alv)
1.Curated_Alv
2.LIM_MAMMARY_LUMINAL_PROGENITOR_UP
3.HOLLERN_PAPILLARY_BREAST_TUMOR
13.KEGG_RIBOSOME
164.CytoTRACE
314.GCS
1048.nCount_RNA
1123.Curated_Hor
20274.percent.mt
22445.Curated_Basal
22541.SMID_BREAST_CANCER_LUMINAL_A_UP
22546.HOLLERN_SQUAMOUS_BREAST_TUMOR
Features by ranking
c
Feature correlation with S1_pseudotime (Hor)
1.Curated_Hor
9.HUPER_BREAST_BASAL_VS_LUMINAL_DN
12.YANG_BREAST_CANCER_ESR1_BULK_UP
13.GO_RESPIRASOME
14.LIM_MAMMARY_LUMINAL_MATURE_UP
500.GCS
1080.CytoTRACE
2311.nCount_RNA
22532.Curated_Basal
22544.SMID_BREAST_CANCER_BASAL_UP
22547.SMID_BREAST_CANCER_LUMINAL_B_DN
22549.LIM_MAMMARY_LUMINAL_MATURE_DN
Features by ranking
d
Curated Stem Gene Sets
S5_pseudotime
Curated Basal Gene Sets
S4_pseudotime
Curated Alv Gene Sets
S2_pseudotime
Curated Hor Gene Sets
S1_pseudotime
GSVA score
Annotation
MaSC/B−pro
Basal
LA−pro
L−Alv
LH−pro
L−Hor
Study
Giraddi
Pal
Bach
Saeki
TabulaMuris

Supplementary Figure 10 Performance evaluation of the curated lineage-specific gene sets. (**a-c**) Correlation of the scGSVA scores for the examined features with S4 pseudotime (Basal, **a**), S2 pseudotime (Alv, **b**), and S1 pseudotime (Hor, **c**). The X-axis represents the rank of the features in terms of the correlation coefficients and the Y-axis represents the correlation coefficients of the scGSVA scores with the pseudotime of interest. The selected features are highlighted and labeled on the plot. The results for the curated gene sets, algorithms, selected RNA-based features, and the cellular characteristics are colored in green, blue, purple, and red, respectively. The numbers on the text labels indicate their rankings. (**d**) The performance of the curated gene sets. The X-axis represents the passage of pseudotime from the node of interest and the Y-axis represents scGSVA scores for the curated gene sets (Stem; 160 genes, Basal; 240 genes, Alv; 500 genes, and Hor; 200 genes) that were individually calculated in each dataset. Each point represents a single cell. The points were color-coded by clusters and datasets. The dashed lines indicate regression lines. Five thousands cells were sampled from the entire data for UMAP visualization.

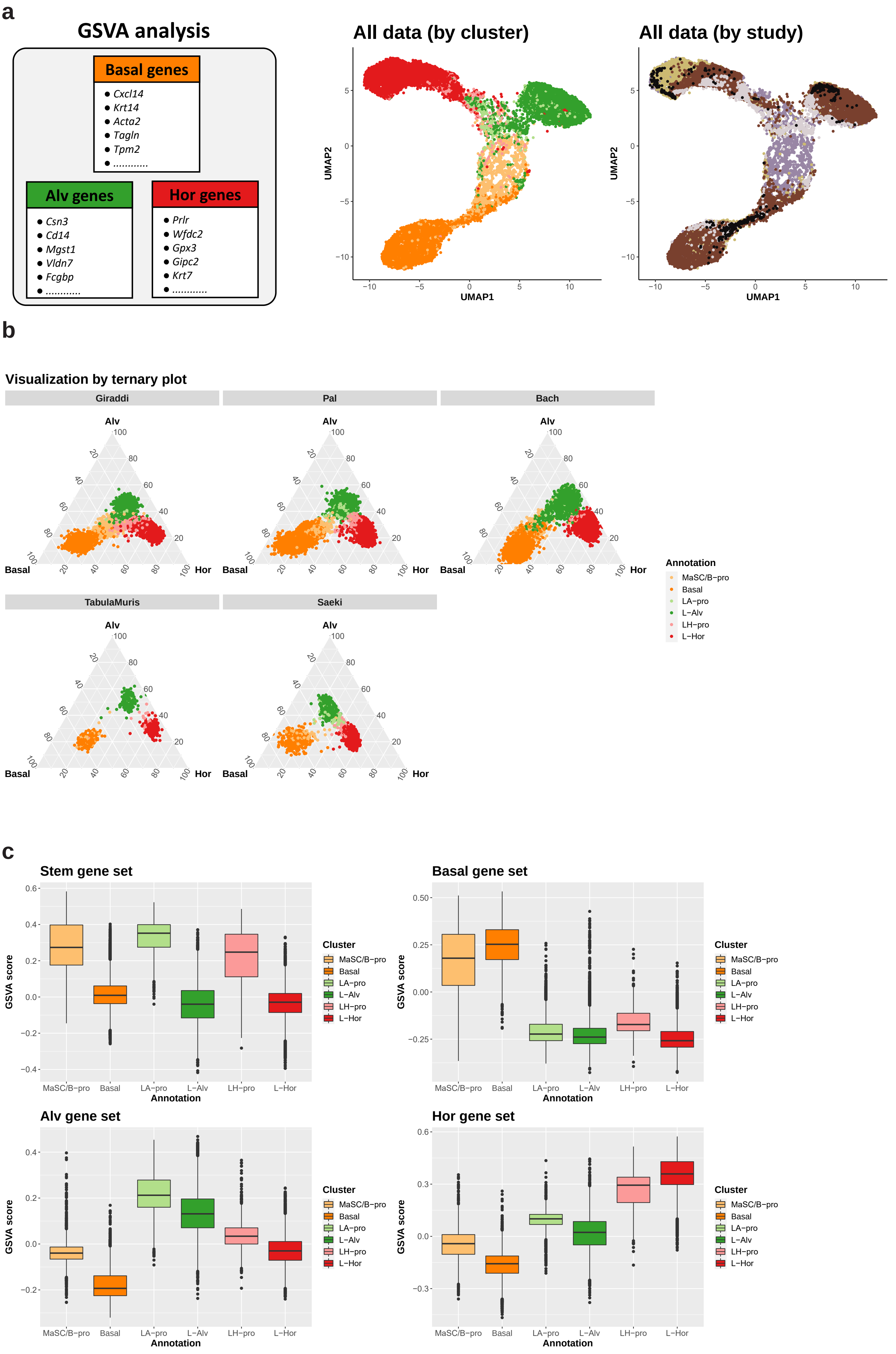
a
GSVA analysis
Basal genes
Cxcl14
Krt14
Acta2
Tagln
Tpm2
....
Alv genes
Csn3
Cd14
Mgst1
Vldn7
Fcgbp
....
Hor genes
Prlr
Wfdc2
Gpx3
Gipc2
Krt7
....
All data (by cluster)
All data (by study)
UMAP1
UMAP2
b
Visualization by ternary plot
Giraddi
Pal
Bach
TabulaMuris
Saeki
Alv
Basal
Hor
Annotation
MaSC/B−pro
Basal
LA−pro
L−Alv
LH−pro
L−Hor
c
Stem gene set
Basal gene set
Alv gene set
Hor gene set
GSVA score
Annotation
Cluster
MaSC/B−pro
Basal
LA−pro
L−Alv
LH−pro
L−Hor

Supplementary Figure 11 Summarization and lineage inference of the scRNAseq data with the curated gene sets. (**a**) The data were summarized with three differentiation-associated gene sets (Basal, Alv, and Hor), and projected on the UMAP plot, color-coded by clusters and datasets. (**b**) Visualization of single cells on ternary plots using the scGSVA scores for the three differentiation-associated gene sets (Basal, Alv, and Hor). (**c**) Distribution of scGSVA scores for the four curated lineage-specific gene sets in each cluster. The n number of each cluster is as follows: MaSC/B-pro (n=4,217), Basal (n=14,493), LA-pro (n=3,176), L-Alv (n=11,856), LH-pro (n=1,156), and L-Hor (n=15,509) (biologically independent samples). The box-plot elements were defined as follows: center line, median; box limits, upper and lower quartiles; whiskers, 1.5x interquartile range; points, outliers.

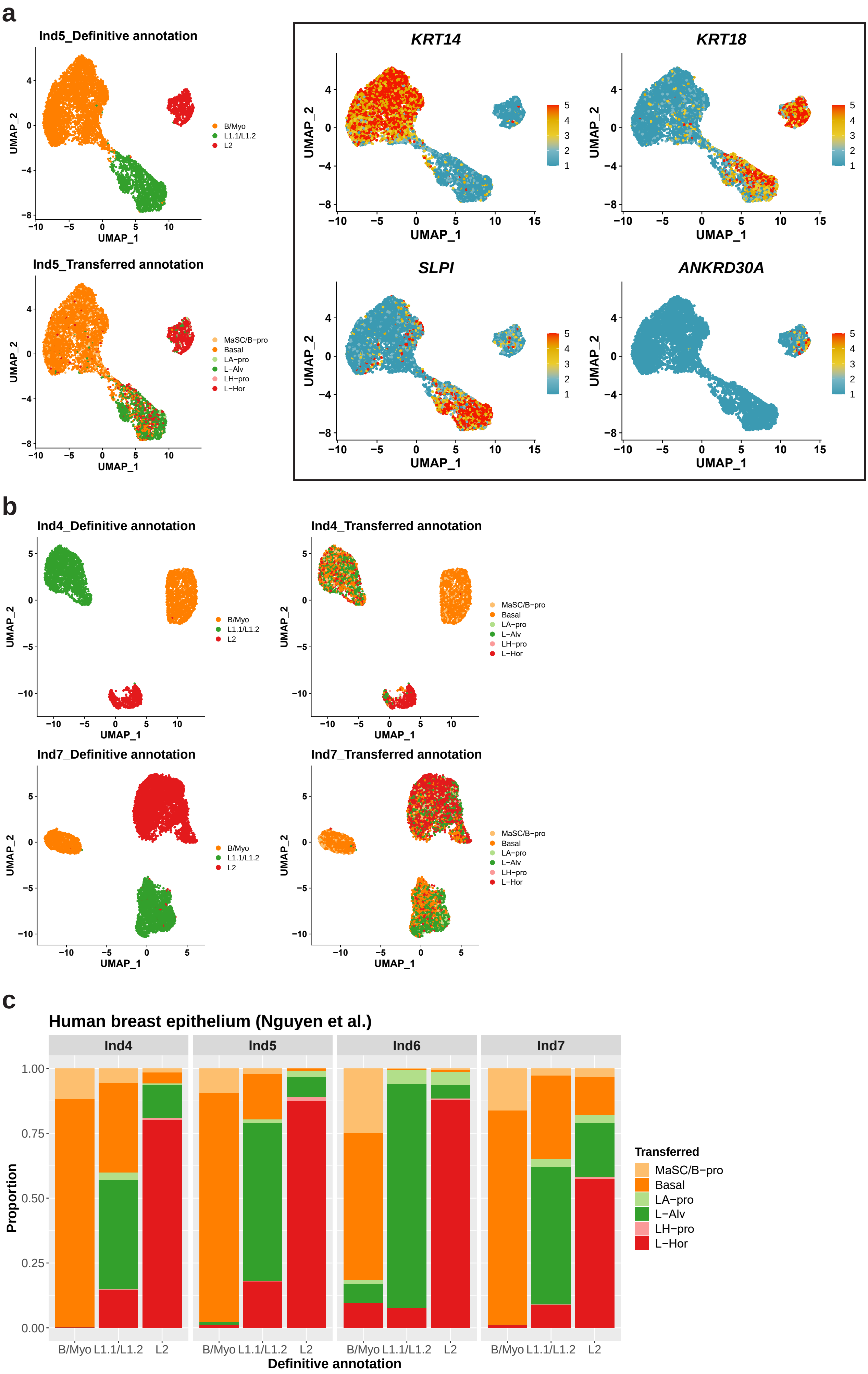

a
Ind5_Definitive annotation
B/Myo
L1.1/L1.2
L2
Ind5_Transferred annotation
MaSC/B−pro
Basal
LA−pro
L−Alv
LH−pro
L−Hor
KRT14
KRT18
SLPI
ANKRD30A
UMAP_1
UMAP_2
b
Ind4_Definitive annotation
Ind4_Transferred annotation
Ind7_Definitive annotation
Ind7_Transferred annotation
c
Human breast epithelium (Nguyen et al.)
Ind4
Ind5
Ind6
Ind7
Proportion
Definitive annotation
Transferred

Supplementary Figure 12 scRNAseq data of the human breast epithelium. (**a**) The definitive and transferred annotation of the cells from individual #five (Ind5). According to the original publication, the expression of marker genes for the definitive annotation was visualized on UMAP plots (*KRT14*; Basal and Myoepithelial cells, *KRT18*; Luminal cells, *SLPI*; L1.1 and L1.2 cells, and *ANKRD30A*; L2). (**b**) The definitive and transferred annotation of the cells from individual #four (Ind4) and individual #seven (Ind7) were projected on UMAP dimensionality. (**c**) The stacked bar plots represent the agreement regarding the lineage inference between the definitive and the transferred annotation, broken down by individuals.

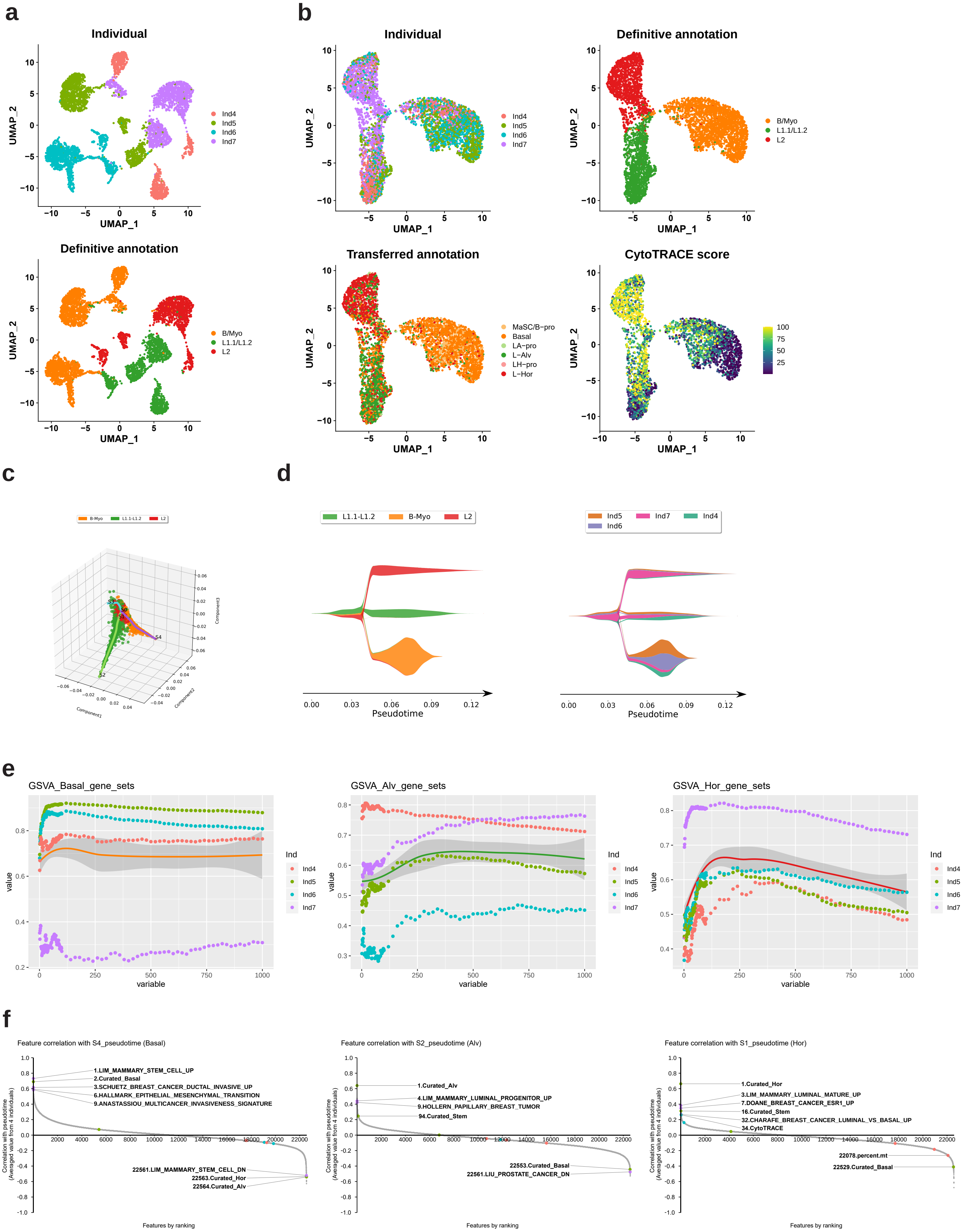
a
Individual
Ind4
Ind5
Ind6
Ind7
UMAP_2
UMAP_1
Definitive annotation
B/Myo
L1.1/L1.2
L2
b
Individual
Definitive annotation
Transferred annotation
MaSC/B−pro
Basal
LA−pro
L−Alv
LH−pro
L−Hor
CytoTRACE score
c
B-Myo
L1.1-L1.2
L2
Component1
Component2
Component3
S0
S1
S2
S3
S4
d
L1.1-L1.2
B-Myo
L2
Ind5
Ind7
Ind4
Ind6
Pseudotime
e
GSVA_Basal_gene_sets
GSVA_Alv_gene_sets
GSVA_Hor_gene_sets
Ind
value
variable
f
Feature correlation with S4_pseudotime (Basal)
1.LIM_MAMMARY_STEM_CELL_UP
2.Curated_Basal
3.SCHUETZ_BREAST_CANCER_DUCTAL_INVASIVE_UP
6.HALLMARK_EPITHELIAL_MESENCHYMAL_TRANSITION
9.ANASTASSIOU_MULTICANCER_INVASIVENESS_SIGNATURE
22561.LIM_MAMMARY_STEM_CELL_DN
22563.Curated_Hor
22564.Curated_Alv
Feature correlation with S2_pseudotime (Alv)
1.Curated_Alv
4.LIM_MAMMARY_LUMINAL_PROGENITOR_UP
9.HOLLERN_PAPILLARY_BREAST_TUMOR
94.Curated_Stem
22553.Curated_Basal
22561.LIU_PROSTATE_CANCER_DN
Feature correlation with S1_pseudotime (Hor)
1.Curated_Hor
3.LIM_MAMMARY_LUMINAL_MATURE_UP
7.DOANE_BREAST_CANCER_ESR1_UP
16.Curated_Stem
32.CHARAFE_BREAST_CANCER_LUMINAL_VS_BASAL_UP
34.CytoTRACE
22078.percent.mt
22529.Curated_Basal
Correlation with pseudotime
(Averaged value from 4 individuals)
Features by ranking

Supplementary Figure 13 Data integration and identification of the lineage-specific gene sets in the human breast epithelium scRNAseq data. (**a**) The merged data, before integration, visualized on UMAP plots and color-coded by individuals and definitive annotation. Five thousands cells were sampled from the entire data for UMAP visualization. (**b**) The integrated data visualized on UMAP plots, colored by individuals, definitive annotation, transferred annotation, and CytoTRACE scores. Five thousands cells were sampled from the entire data for UMAP visualization. (**c**) The trajectory learned in the Mlle space using 1,000 human breast epithelial cells from each individual. (**d**) The stream plots of the 24K human breast epithelial cells, color-coded by definitive clusters and individuals. (**e**) The change in the performance of the gene sets according to the number of the top correlated genes in a gene set. The X-axis represents the number of gene(s) in a gene set and the Y-axis represents the correlation between the scGSVA score and the pseudotime [S4; Basal, S2; Alv, and S1; Hor]. The correlation was calculated on an individual basis. The solid lines represent regression curves in a LOESS model and the surrounding grey areas represent the confidence intervals. (**f**) Correlation of the scGSVA scores for the examined features with S4 pseudotime (Basal), S2 pseudotime (Alv), and S1 pseudotime (Hor). The X-axis represents the rank of the features in terms of the correlation coefficients and the Y-axis represents the correlation coefficients of the scGSVA scores with the pseudotime of interest. The selected features are highlighted and labeled on the plot. The results for the curated gene sets, algorithms, selected RNA-based features, and cellular characteristics are colored in green, blue, purple, and red, respectively. The numbers on the text labels indicate the ranking of features.

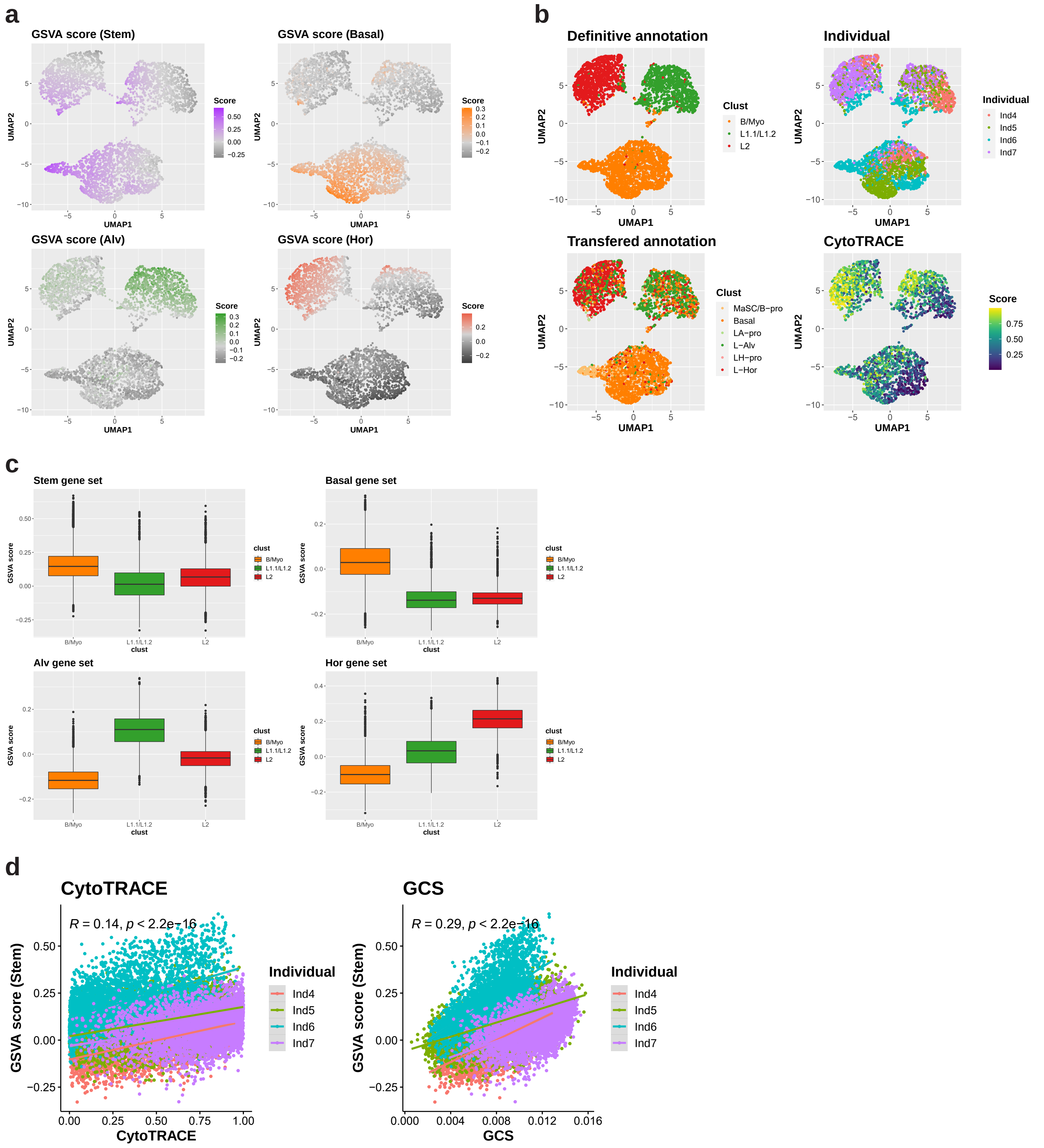

a
GSVA score (Stem)
GSVA score (Basal)
GSVA score (Alv)
GSVA score (Hor)
Score
UMAP1
UMAP2
b
Definitive annotation
Individual
Transfered annotation
CytoTRACE
Clust
B/Myo
L1.1/L1.2
L2
Ind4
Ind5
Ind6
Ind7
MaSC/B−pro
Basal
LA−pro
L−Alv
LH−pro
L−Hor
c
Stem gene set
Basal gene set
Alv gene set
Hor gene set
GSVA score
clust
d
CytoTRACE
GCS
R = 0.14, p < 2.2e−16
R = 0.29, p < 2.2e−16
GSVA score (Stem)

Supplementary Figure 14 Lineage-specific gene sets-based summarization of the human breast epithelium scRNAseq data. (**a**) The scGSVA scores for the four curated gene sets used for summarization were projected on UMAP plots. Five thousands cells were sampled from the entire data for UMAP visualization. (**b**) UMAP plots colored by definitive annotation, individuals, transferred annotation, and CytoTRACE scores. Five thousands cells were sampled from the entire data for UMAP visualization. (**c**) Distribution of the scGSVA scores for the four curated lineage-specific gene sets in each cluster. The n number of each cluster is as follows: B/Myo (n=11,205), L1.1/L1.2 (n=6,687), and L2 (n=6,388) (biologically independent samples). The box-plot elements were defined as follows: center line, median; box limits, upper and lower quartiles; whiskers, 1.5x interquartile range; points, outliers. (**d**) The correlations of the scGSVA scores for the curated Stem gene set with the CytoTRACE score and Gene Count Signature (GCS). n = 14,967 cells, Spearman's rank correlation.

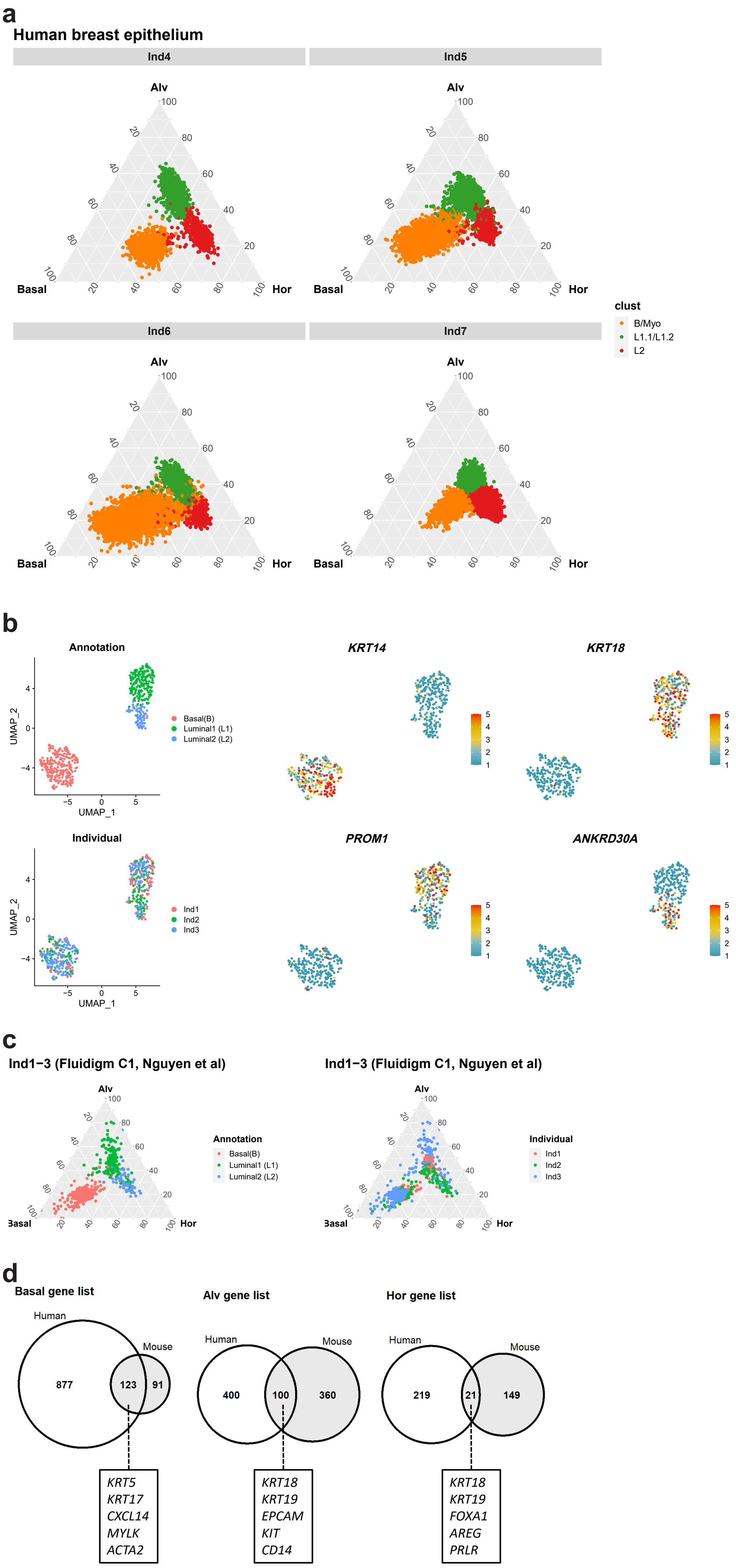
a
Human breast epithelium
Ind4
Ind5
Ind6
Ind7
Alv
Basal
Hor
clust
B/Myo
L1.1/L1.2
L2
b
Annotation
UMAP_2
UMAP_1
Basal(B)
Luminal1 (L1)
Luminal2 (L2)
KRT14
KRT18
Individual
Ind1
Ind2
Ind3
PROM1
ANKRD30A
c
Ind1−3 (Fluidigm C1, Nguyen et al)
Ind1−3 (Fluidigm C1, Nguyen et al)
Annotation
Basal(B)
Luminal1 (L1)
Luminal2 (L2)
Individual
Ind1
Ind2
Ind3
d
Basal gene list
Human
Mouse
877
123
91
KRT5
KRT17
CXCL14
MYLK
ACTA2
Alv gene list
Human
Mouse
400
100
360
KRT18
KRT19
EPCAM
KIT
CD14
Hor gene list
Human
Mouse
219
21
149
KRT18
KRT19
FOXA1
AREG
PRLR

Supplementary Figure 15 Application of the human lineage gene sets to the additional datasets and the comparison between the species. (**a**) Visualization of the single cells on ternary plots using the scGSVA scores for the three differentiation-associated gene sets (Basal, Alv, and Hor). The four individuals from the 10X data were shown. (**b**) The additional three individuals from the Fluidigm C1 data visualized on UMAP plots and color-coded by definitive annotation and individuals. According to the original publication, the expression of marker genes for the definitive annotation was visualized on UMAP plots (*KRT14*; Basal and Myoepithelial cells, *KRT18*; Luminal cells, *PROM1*; L1 cells, and *ANKRD30A*; L2 cells). (**c**) Visualization of additional data on ternary plots based on the scGSVA scores for the three differentiation-associated gene sets (Basal, Alv, and Hor). The plots were color-coded by definitive annotation and individuals. (**d**) Comparisons of the lineage gene sets between mice and humans. The representative common genes were shown.

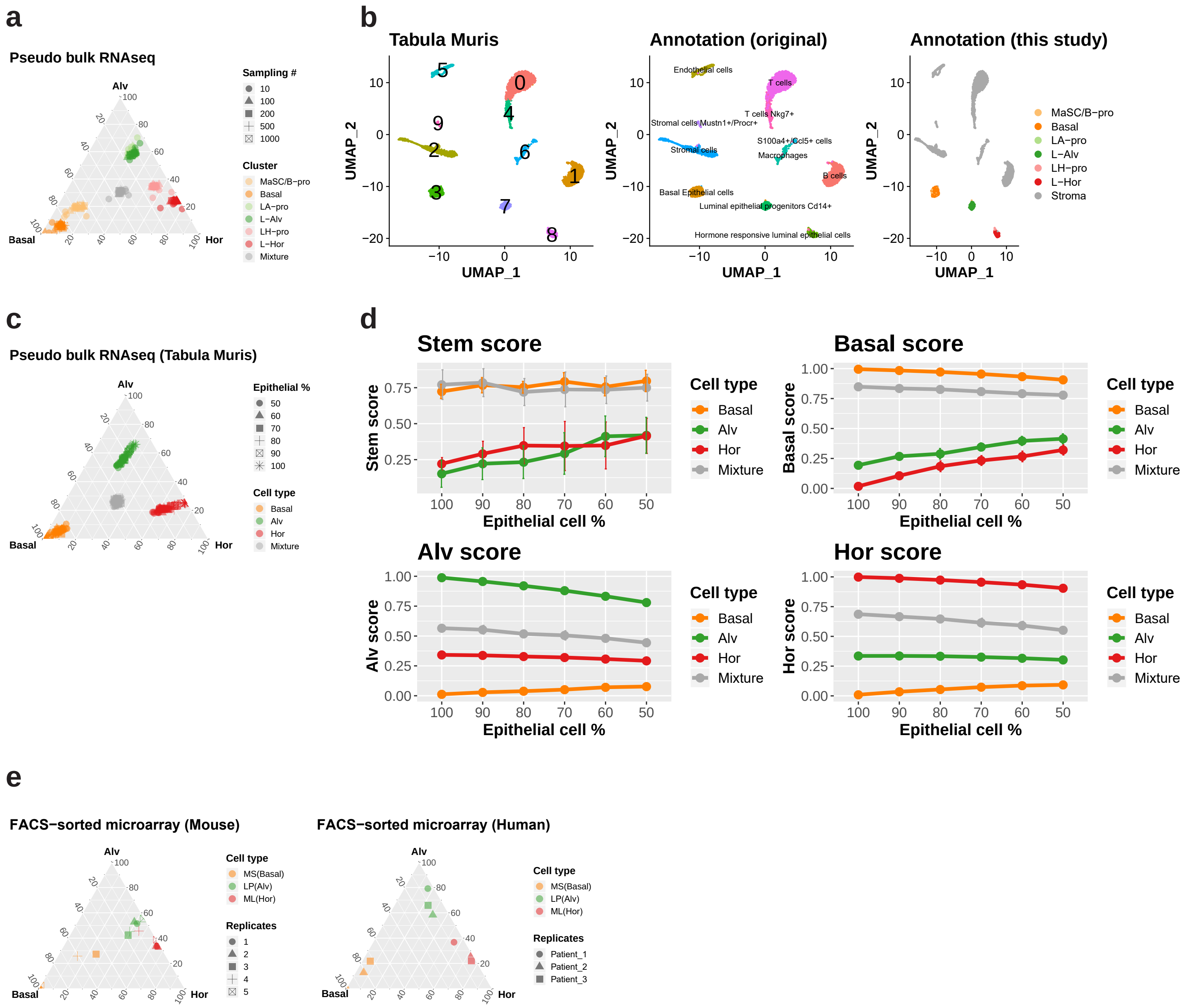
a
Pseudo bulk RNAseq
Alv
Basal
Hor
Sampling #
10
100
200
500
1000
Cluster
MaSC/B−pro
Basal
LA−pro
L−Alv
LH−pro
L−Hor
Mixture
b
Tabula Muris
Annotation (original)
Annotation (this study)
UMAP_1
UMAP_2
Endothelial cells
T cells
T cells Nkg7+
Stromal cells Mustn1+/Procr+
Stromal cells
S100a4+/Ccl5+ cells
Macrophages
B cells
Basal Epithelial cells
Luminal epithelial progenitors Cd14+
Hormone responsive luminal epithelial cells
MaSC/B−pro
Basal
LA−pro
L−Alv
LH−pro
L−Hor
Stroma
c
Pseudo bulk RNAseq (Tabula Muris)
Epithelial %
50
60
70
80
90
100
Cell type
Basal
Alv
Hor
Mixture
d
Stem score
Basal score
Alv score
Hor score
Epithelial cell %
Cell type
Basal
Alv
Hor
Mixture
e
FACS−sorted microarray (Mouse)
FACS−sorted microarray (Human)
Cell type
MS(Basal)
LP(Alv)
ML(Hor)
Replicates
1
2
3
4
5
Patient_1
Patient_2
Patient_3

Supplementary Figure 16 Extention of the lineage inference to the bulk RNAseq data. (**a**) The pseudo bulk RNA seq of the different cell types in the mouse mammary epithelium with different sampling numbers from the scRNAseq data. Mixture indicates that cells were evenly sampled from the six different cell types. (**b**) The Tabula Muris data used for the pseudo tissue bulk RNAseq. The Louvain clustering, original annotations in the project, and clustering of the epithelial cells obtained from the integrative analysis were visualized on UMAP plots. (**c**) The pseudo bulk RNA seq of the three different cell lineages in the mouse mammary epithelium mixed with the varying number of stromal cells. Mixture indicates that cells were evenly sampled from the three different lineages. (**d**) The changes in the scGSVA scores for the curated lineage-specific gene sets, with the increasing proportion of the stromal cells in the pseudo bulk RNAseq. Each data was generated by averaging the results from 10 independent simulations. Error bars represent standard deviations. (**e**) The FACS-sorted transcriptome analysis of the three major cell types in the mouse and human mammary epithelium. The results were scored using the scGSVA analysis with the lineage-specific gene sets

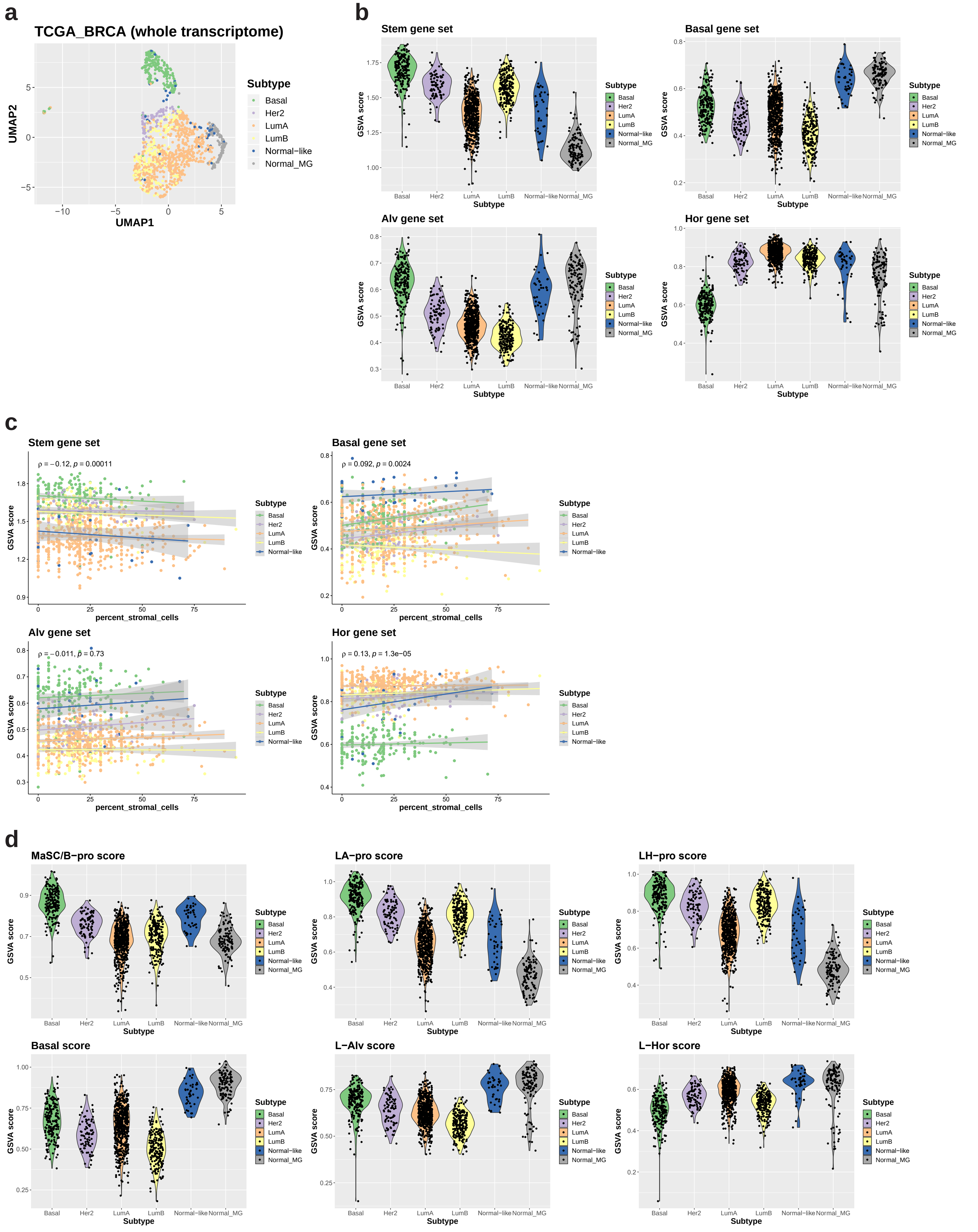
a
TCGA_BRCA (whole transcriptome)
UMAP1
UMAP2
Subtype
Basal
Her2
LumA
LumB
Normal−like
Normal_MG
b
Stem gene set
Basal gene set
Alv gene set
Hor gene set
GSVA score
Subtype
c
Stem gene set
ρ = −0.12, p = 0.00011
Basal gene set
ρ = 0.092, p = 0.0024
Alv gene set
ρ = −0.011, p = 0.73
Hor gene set
ρ = 0.13, p = 1.3e−05
percent_stromal_cells
GSVA score
d
MaSC/B−pro score
LA−pro score
LH−pro score
Basal score
L−Alv score
L−Hor score
GSVA score
Subtype

Supplementary Figure 17 scGSVA analysis of the TCGA BRCA data. (**a**) The summarization of the data in UMAP dimensionality, considering the expression of all detected transcripts. (**b**) Distribution of the scGSVA scores for the four curated lineage-specific gene sets in each subtype. (**c**) The correlation of the scGSVA scores for the lineage-specific gene sets with the stromal proportion in the tumor tissue (n=1,083). Spearman's rank correlation. (**d**) Distribution of the scGSVA scores for the DEGs of the six clusters identified in the mouse integrated data in each subtype. The n number of each subtype is as follows: Basal (n=194), Her2 (n=82), LumA (n=567), LumB (n=207), Normal-like (n=40), and Normal_MG (n=113) (biologically independent samples).

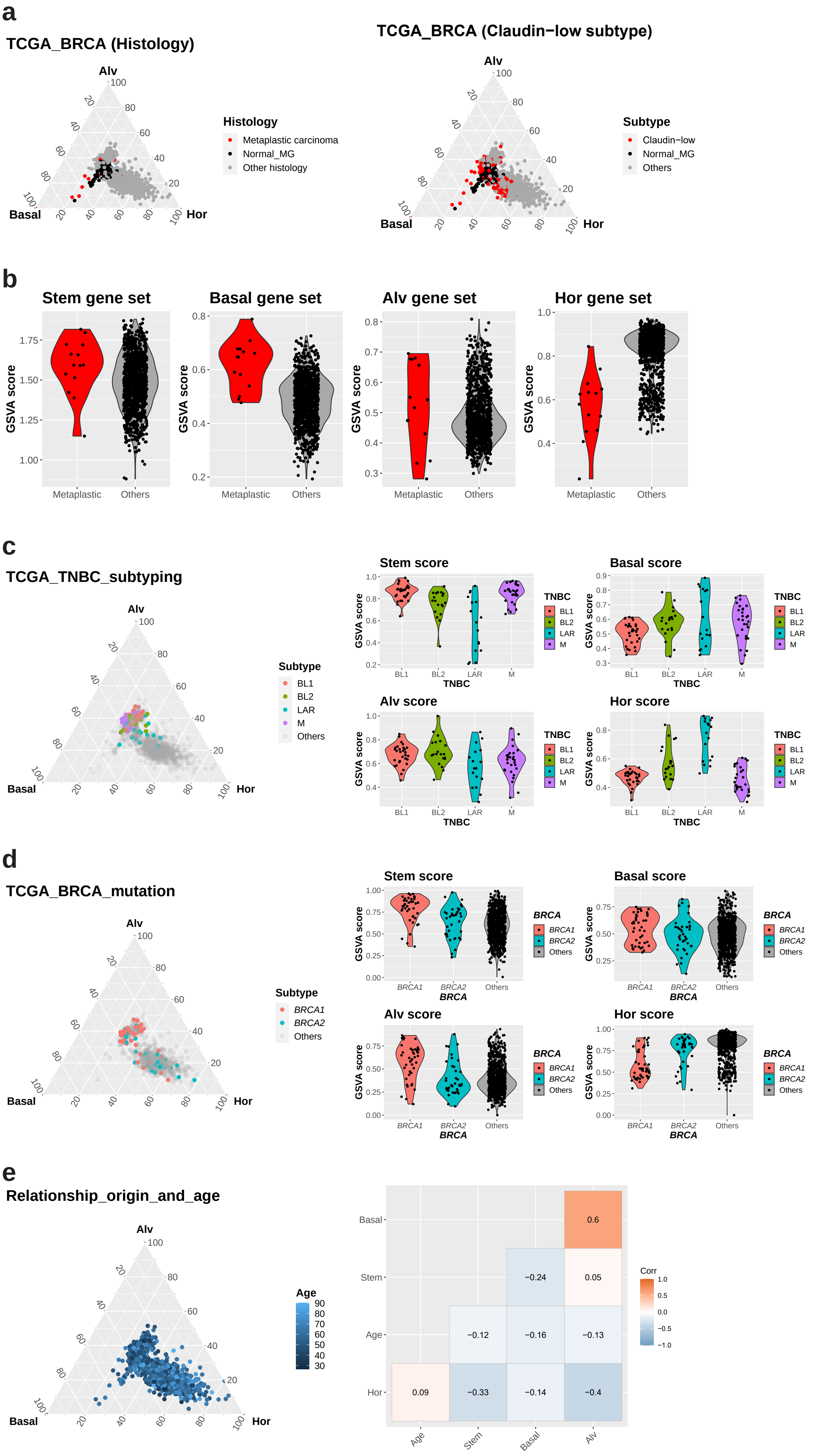

a
TCGA_BRCA (Histology)
TCGA_BRCA (Claudin−low subtype)
Alv
Basal
Hor
Histology
Metaplastic carcinoma
Normal_MG
Other histology
Subtype
Claudin−low
Normal_MG
Others
b
Stem gene set
Basal gene set
Alv gene set
Hor gene set
GSVA score
Metaplastic
Others
c
TCGA_TNBC_subtyping
Subtype
BL1
BL2
LAR
M
Others
Stem score
Basal score
Alv score
Hor score
TNBC
d
TCGA_BRCA_mutation
BRCA1
BRCA2
BRCA
e
Relationship_origin_and_age
Age
Basal
Stem
Age
Hor
Alv
Corr
0.6
−0.24
0.05
−0.12
−0.16
−0.13
0.09
−0.33
−0.14
−0.4

Supplementary Figure 18 Breakdown of the TCGA data by additional features. (**a**) The ternary plots of the TCGA data for the three differentiation gene sets. Metaplastic carcinomas and Claudin-low tumors were highlighted in red. (**b**) The comparisons of the scores for the four lineage-specific gene sets between metaplastic carcinomas (n=14) and the other histologies (n=1,075) (biologically independent samples). (**c**) The TNBCtype-4 subtypes. Distribution of the subtypes were overlaid on the ternary plot and the scGSVA scores were broken down by subtypes. The n number of each subtype is as follows: BL1 (n=30), BL2 (n=20), LAR (n=17), and M (n=28) (biologically independent samples). (**d**) The *BRCA* status. The *BRCA* status were overlaid on the ternary plot and the scGSVA scores were broken down by status. The n number of each group is as follows: BRCA1 (n=42), BRCA2 (n=37), and Others (n=767) (biologically independent samples). (**e**) The relathonship of scGSVA scores with age at diagnosis. Age at diagnosis was overlaid on the ternary plot and correlation analysis was performed with each scGSVA score.

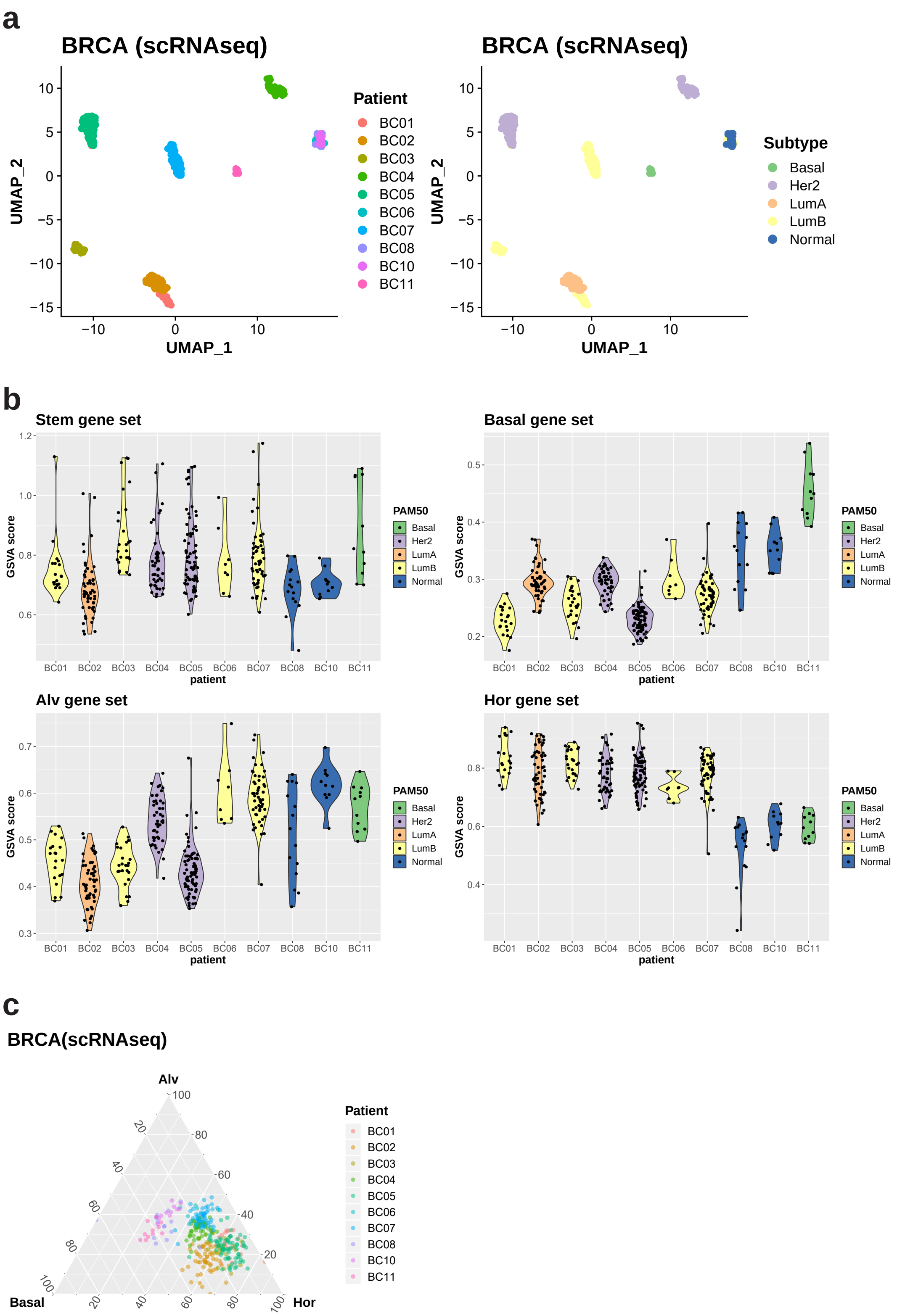
a
BRCA (scRNAseq)
UMAP_1
UMAP_2
Patient
BC01
BC02
BC03
BC04
BC05
BC06
BC07
BC08
BC10
BC11
BRCA (scRNAseq)
Subtype
Basal
Her2
LumA
LumB
Normal
b
Stem gene set
Basal gene set
Alv gene set
Hor gene set
GSVA score
patient
PAM50
Basal
Her2
LumA
LumB
Normal
c
BRCA(scRNAseq)
Alv
Basal
Hor
Patient
BC01
BC02
BC03
BC04
BC05
BC06
BC07
BC08
BC10
BC11

Supplementary Figure 19 scRNAseq data of human breast cancer. (**a**) UMAP plots of the human breast cancer scRNAseq data colored by patients and subtypes. (**b**) Distribution of the scGSVA scores for the four curated lineage-specific gene sets in each patient. The cell number of each patient is as follows: BC01 (n=20), BC02 (n=53), BC03 (n=25), BC04 (n=47), BC05 (n=75), BC06 (n=8), BC07 (n=52), BC08 (n=15), BC10 (n=11), and BC11 (n=11) (biologically independent samples). (**c**) The ternary plot of the human breast cancer scRNAseq data for the three differentiation gene sets, color-coded by patients.

a

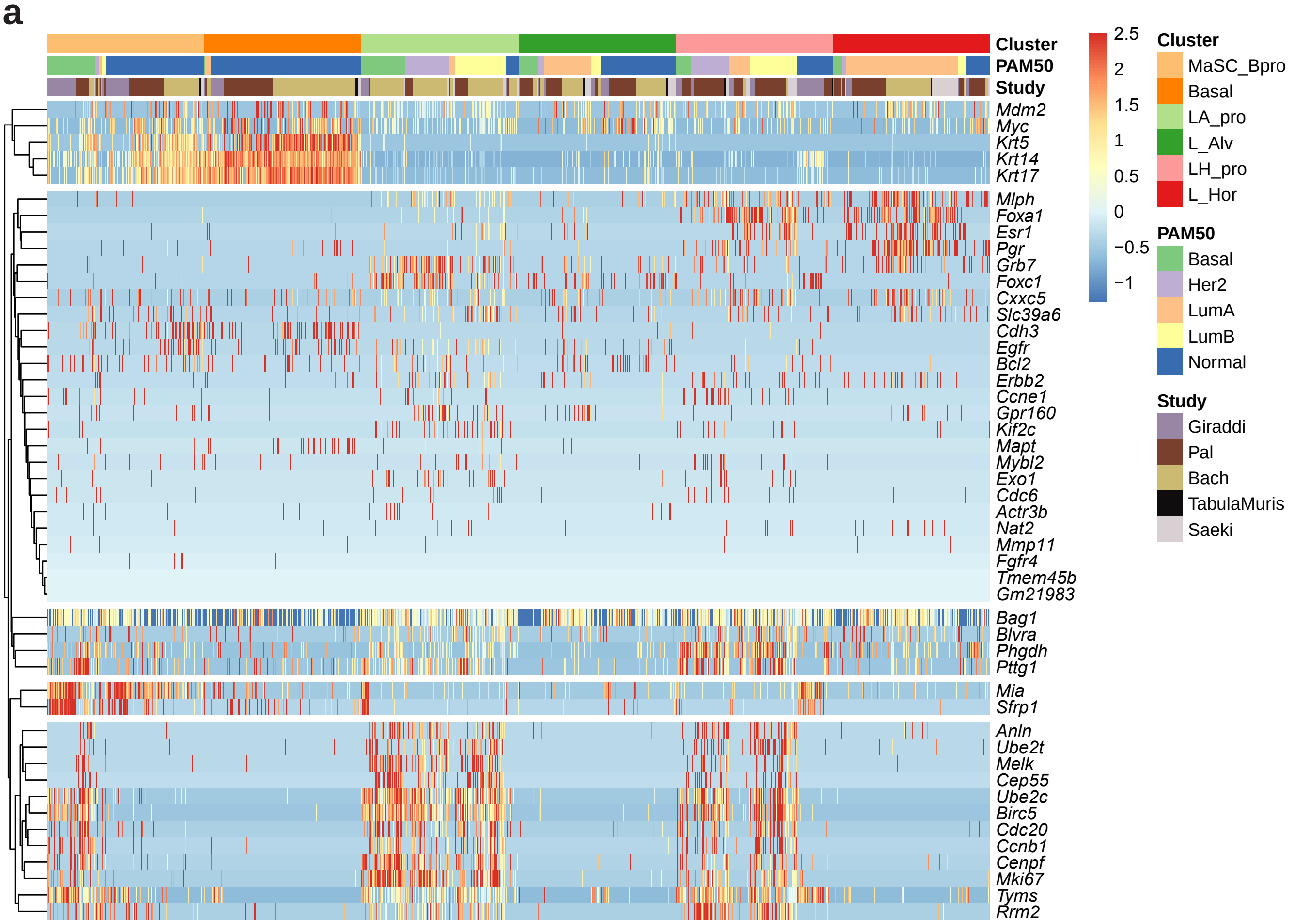

Cluster
PAM50
Study
Mdm2
Myc
Krt5
Krt14
Krt17
Mlph
Foxa1
Esr1
Pgr
Grb7
Foxc1
Cxxc5
Slc39a6
Cdh3
Egfr
Bcl2
Erbb2
Ccne1
Gpr160
Kif2c
Mapt
Mybl2
Exo1
Cdc6
Actr3b
Nat2
Mmp11
Fgfr4
Tmem45b
Gm21983
Bag1
Blvra
Phgdh
Pttg1
Mia
Sfrp1
Anln
Ube2t
Melk
Cep55
Ube2c
Birc5
Cdc20
Ccnb1
Cenpf
Mki67
Tyms
Rrm2
2.5
2
1.5
1
0.5
0
−0.5
−1
Cluster
MaSC_Bpro
Basal
LA_pro
L_Alv
LH_pro
L_Hor
PAM50
Basal
Her2
LumA
LumB
Normal
Study
Giraddi
Pal
Bach
TabulaMuris
Saeki

b

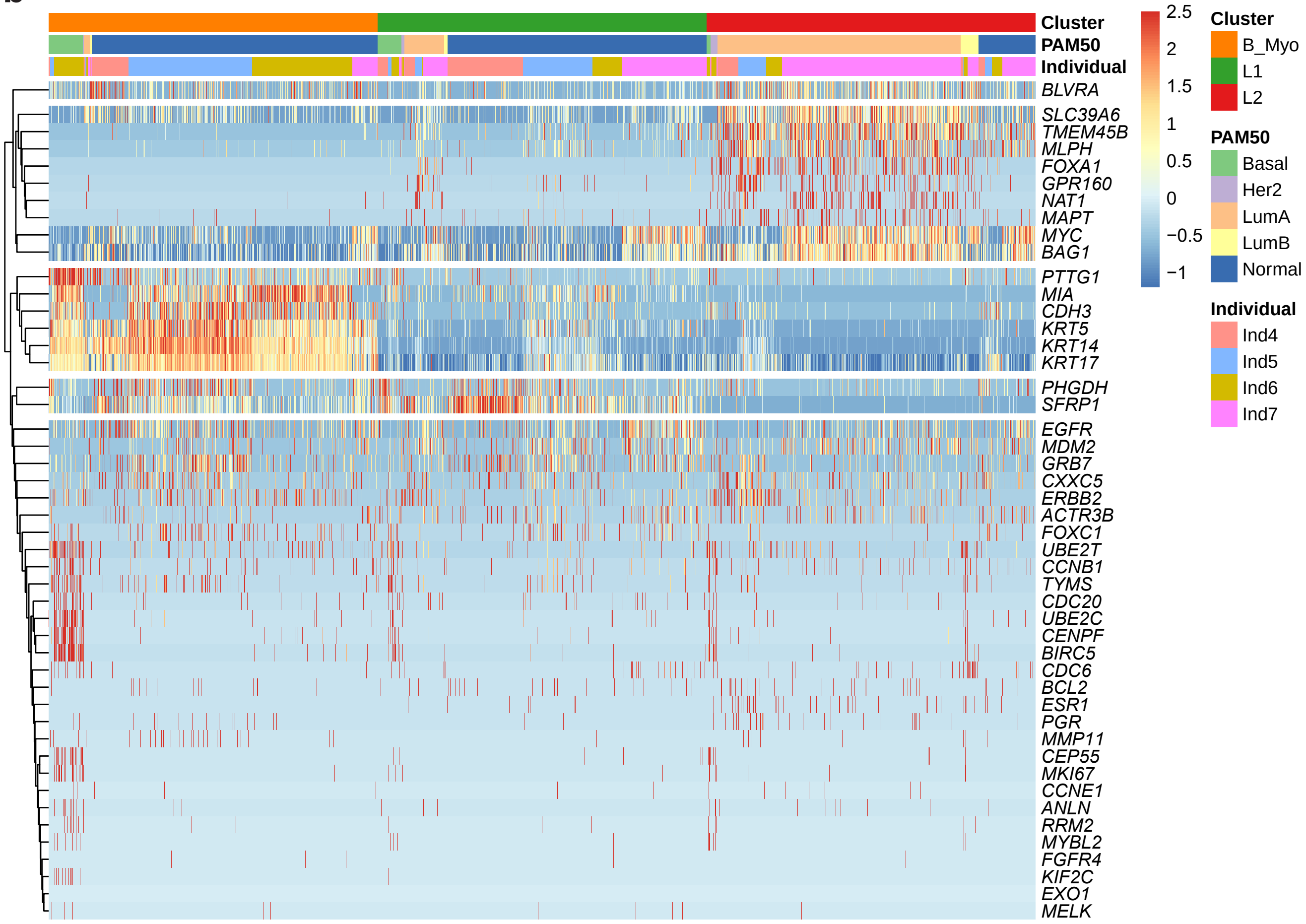

Cluster
PAM50
Individual
BLVRA
SLC39A6
TMEM45B
MLPH
FOXA1
GPR160
NAT1
MAPT
MYC
BAG1
PTTG1
MIA
CDH3
KRT5
KRT14
KRT17
PHGDH
SFRP1
EGFR
MDM2
GRB7
CXXC5
ERBB2
ACTR3B
FOXC1
UBE2T
CCNB1
TYMS
CDC20
UBE2C
CENPF
BIRC5
CDC6
BCL2
ESR1
PGR
MMP11
CEP55
MKI67
CCNE1
ANLN
RRM2
MYBL2
FGFR4
KIF2C
EXO1
MELK
2.5
2
1.5
1
0.5
0
−0.5
−1
Cluster
B_Myo
L1
L2
PAM50
Basal
Her2
LumA
LumB
Normal
Individual
Ind4
Ind5
Ind6
Ind7

Supplementary Figure 20 Reflection of the PAM50 molecular subtyping on the scRNAseq data of the mammary epithelium. The heatmaps visualized the expression of the genes used for the PAM50 classification grouped by clusters, subtypes, and datasets. (**a**) Mouse normal mammary epithelium. (**b**) Human normal breast epithelium.

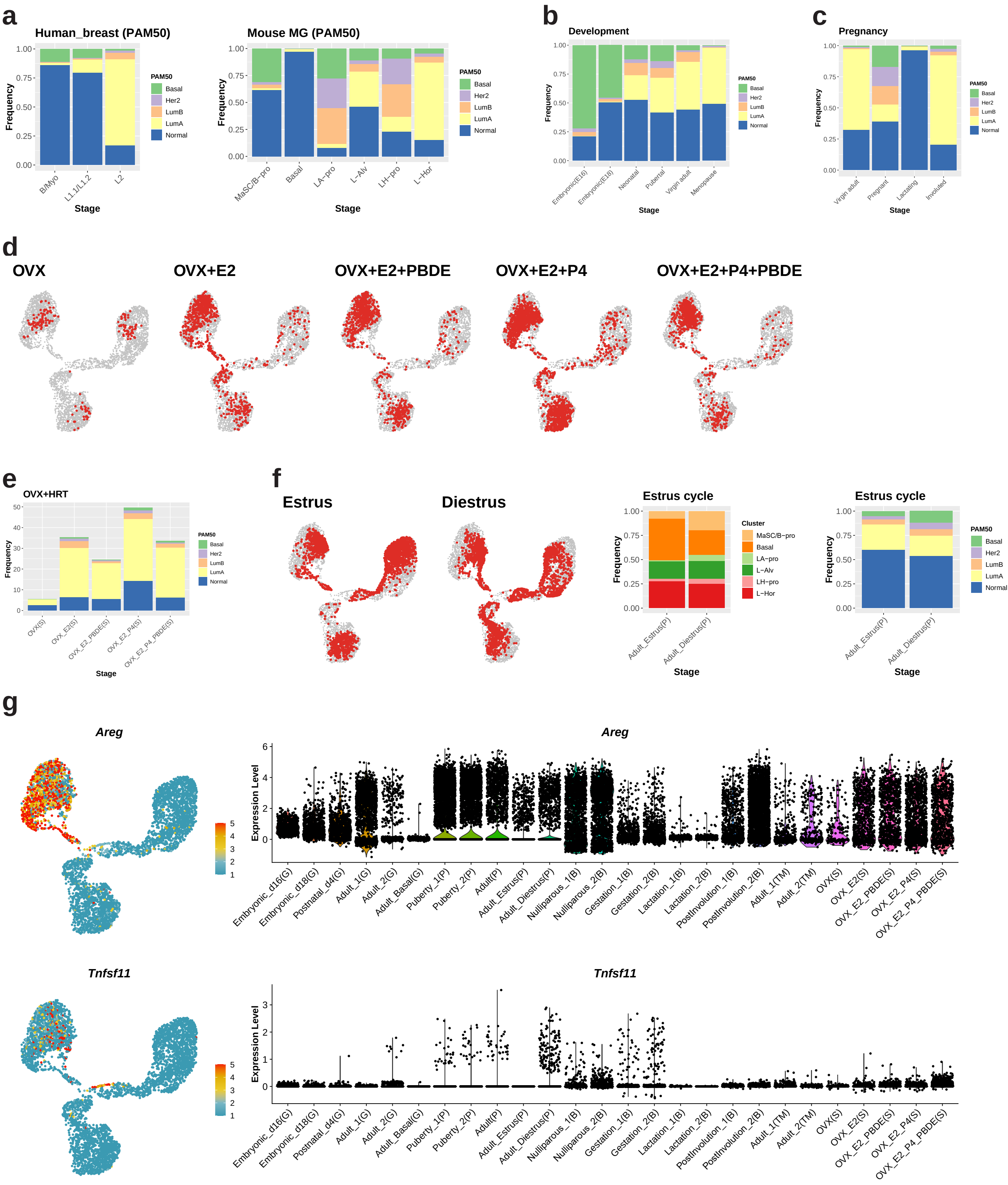

a
Human_breast (PAM50)
Mouse MG (PAM50)
Frequency
Stage
B/Myo
L1.1/L1.2
L2
MaSC/B-pro
Basal
LA-pro
L-Alv
LH-pro
L-Hor
PAM50
Basal
Her2
LumB
LumA
Normal
b
Development
Embryonic(E16)
Embryonic(E18)
Neonatal
Pubertal
Virgin adult
Menopause
c
Pregnancy
Virgin adult
Pregnant
Lactating
Involuted
d
OVX
OVX+E2
OVX+E2+PBDE
OVX+E2+P4
OVX+E2+P4+PBDE
e
OVX+HRT
OVX(S)
OVX_E2(S)
OVX_E2_PBDE(S)
OVX_E2_P4(S)
OVX_E2_P4_PBDE(S)
f
Estrus
Diestrus
Estrus cycle
Cluster
MaSC/B−pro
Basal
LA−pro
L−Alv
LH−pro
L−Hor
Adult_Estrus(P)
Adult_Diestrus(P)
g
Areg
Tnfsf11
Expression Level
Embryonic_d16(G)
Embryonic_d18(G)
Postnatal_d4(G)
Adult_1(G)
Adult_2(G)
Adult_Basal(G)
Puberty_1(P)
Puberty_2(P)
Adult(P)
Adult_Estrus(P)
Adult_Diestrus(P)
Nulliparous_1(B)
Nulliparous_2(B)
Gestation_1(B)
Gestation_2(B)
Lactation_1(B)
Lactation_2(B)
PostInvolution_1(B)
PostInvolution_2(B)
Adult_1(TM)
Adult_2(TM)
OVX(S)
OVX_E2(S)
OVX_E2_PBDE(S)
OVX_E2_P4(S)
OVX_E2_P4_PBDE(S)

Supplementary Figure 21 Reorganization of the gland during development, pregnancy, menopausal HRT with exposure to PBDEs, and estrus cycle and its implication for the risk of specific breast cancer subtypes. (**a**) The results of the PAM50 subtyping of each cluster in the normal human and mouse mammary epithelium. (**b**) Distribution of PAM50 classification at different life stages. (**c**) Distribution of PAM50 classification during pregnancy. (**d**) The reorganization of the mammary gland by the HRTs and the PBDEs exposure in the surgically menopaused mice. The cells from each sample were highlighted in red on the UMAP plots. Five thousands cells were sampled from the entire data for background UMAP visualization. (**e**) The stacked bar plot represents the proportion of the epithelial cells in the entire gland, color-coded by the PAM50 classification. (**f**) The mammary gland reorganization during the estrus cycle. The cells from each sample were highlighted on the UMAP dimensionality. The proportion of each cluster and PAM50 subtype in the mammary epithelium is summarized in the stacked bar plots. Five thousands cells were sampled from the entire data for background UMAP visualization. (**g**) Expression of the soluble RNA messengers in the mammary gland and their fluctuations. The expression of amphiregulin (*Areg*) and RANKL (*Tnfsf11*) were visualized on UMAP plots and violin plots, grouped by samples. The n number of each sample is summarized in Supplementary Data 2. HRT; hormone replacement therapy. HRT: hormone replacement therapy.

Supplementary References

1. Anaconda Software Distribution. Anaconda. https://anaconda.com (2020).

2. Huber, W. *et al.* Orchestrating high-throughput genomic analysis with Bioconductor. *Nat. Methods* 12, 115–121 (2015).

3. Durinck, S., Spellman, P. T., Birney, E. & Huber, W. Mapping Identifiers for the Integration of Genomic Datasets with the R/Bioconductor package biomaRt. *Nat. Protoc.* 4, 1184–1191 (2009).

4. Yu, G., Wang, L.-G., Han, Y. & He, Q.-Y. clusterProfiler: an R Package for Comparing Biological Themes Among Gene Clusters. *OMICS* 16, 284–287 (2012).

5. Wilke, C. cowplot: Streamlined Plot Theme and Plot Annotations for 'ggplot2'. R package version 1.0.0. https://CRAN.R-project.org/package=cowplot (2019).

6. Gulati, G. S. *et al.* Single-cell transcriptional diversity is a hallmark of developmental potential. *Science* 367, 405–411 (2020).

7. McGinnis, C. S., Murrow, L. M. & Gartner, Z. J. DoubletFinder: Doublet Detection in Single-Cell RNA Sequencing Data Using Artificial Nearest Neighbors. *Cell Syst.* **8**, 329–337.e4; 10.1016/j.cels.2019.03.003 (2019).

8. Wickham, H., François, R., Henry, L. & Müller, K. dplyr: A Grammar of Data Manipulation. R package version 0.8.3. https://CRAN.R-project.org/package=dplyr (2019).

9. Lê, S., Josse, J. & Husson, F. FactoMineR: An R Package for Multivariate Analysis. *J. Stat. Softw.* 25, 1–18 (2008).

10. Kassambara, A. & Mundt, F. factoextra: Extract and Visualize the Results of Multivariate Data Analyses. R package version 1.0.5. https://CRAN.R-project.org/package=factoextra (2017).

11. Gendoo, D. M. A. *et al.* Genefu: an R/Bioconductor package for computation of gene expression-based signatures in breast cancer. *Bioinformatics* 32, 1097–1099 (2016).

12. Kassambara, A. ggcorrplot: Visualization of a Correlation Matrix using 'ggplot2'. R package version 0.1.3. https://CRAN.R-project.org/package=ggcorrplot (2019).

13. Wickham, H. *ggplot2: Elegant Graphics for Data Analysis*. (Springer-Verlag, 2009).

14. Slowikowski, K. ggrepel: Automatically Position Non-Overlapping Text Labels with 'ggplot2'. R package version 0.8.1. https://CRAN.R-project.org/package=ggrepel (2019)..

15. Kassambara, A. ggpubr: 'ggplot2' Based Publication Ready Plots. R package version 0.2.3. https://CRAN.R-project.org/package=ggpubr (2019).

16. Hamilton, N. E. & Ferry, M. ggtern: Ternary Diagrams Using ggplot2. *J. Stat. Softw.* 87, 1–17 (2018).

17. Hänzelmann, S., Castelo, R. & Guinney, J. GSVA: gene set variation analysis for microarray and RNA-Seq data. *BMC Bioinformatics* **14**, 7; 10.1186/1471-2105-14-7 (2013).

18. Thomas, K. *et al.* Jupyter Notebooks - a publishing format for reproducible computational workflows in *Positioning and Power in Academic Publishing: Players, Agents and Agendas (eds. Loizides, F. and Scmidt, B.)* 87–90 (IOS Press, 2016).

19. Korsunsky, I. *et al.* Fast, sensitive and accurate integration of single-cell data with Harmony. *Nat. Methods* **16**, 1289–1296 (2019).

20. Liu, J. *et al.* Jointly defining cell types from multiple single-cell datasets using LIGER. *Nat. Protoc.* 15, 3632–3662 (2020).

21. Hunter, J. D. Matplotlib: A 2D Graphics Environment. *Comput. Sci. Eng.* 9, 90–95 (2007).

22. Dolgalev, I. msigdbr: MSigDB Gene Sets for Multiple Organisms in a Tidy Data Format. R package version 7.0.1. https://CRAN.R-project.org/package=msigdbr (2019).

23. Oliphant, T. E. *Guide to NumPy: 2nd Edition*. (Continuum Press, 2015).

24. McKinney, W. Data Structures for Statistical Computing in Python. In: Proceedings of the 9th Python in Science Conference, Volume 445, 2010.

25. Kolde, R. pheatmap: Pretty Heatmaps. R package version 1.0.12. https://CRAN.R-project.org/package=pheatmap (2019).

26. Sievert, C. *Interactive Web-Based Data Visualization with R, plotly, and shiny*. (CRC Press, 2020).

27. Rossum, G. V. & Drake, F. L. *Python 3 Reference Manual.* (CreateSpace Independent Publishing Platform, 2009).

28. R Core Team. R: A language and environment for statistical computing. http://www.R-project.org/ (2013).

29. Wickham, H. Reshaping Data with the reshape Package. *J. Stat. Softw.* 21, 1–20 (2007).

30. RStudio Team. RStudio: Integrated Development for R. http://www.rstudio.com/ (2020).

31. Johansen, N. & Quon, G. scAlign: a tool for alignment, integration, and rare cell identification from scRNA-seq data. *Genome Biol.* **20**, 166; 10.1186/s13059-019-1766-4 (2019).

32. Stuart, T. *et al.* Comprehensive Integration of Single-Cell Data. *Cell* 177, 1888-1902.e21 (2019).

33. Chen, H. *et al.* Single-cell trajectories reconstruction, exploration and mapping of omics data with STREAM. *Nat. Commun.* **10**, 1903; 10.1038/s41467-019-09670-4 (2019).

34. Wickham, H. stringr: Simple, Consistent Wrappers for Common String Operations. R package version 1.4.0. https://CRAN.R-project.org/package=stringr (2019).

35. Morgan M., Obenchain, V., Hester, J. & Pagès, H. SummarizedExperiment: SummarizedExperiment container. R package version 1.14.1. https://bioconductor.org/packages/SummarizedExperiment (2019).

36. Colaprico, A. *et al.* TCGAbiolinks: an R/Bioconductor package for integrative analysis of TCGA data. *Nucleic Acids Res.* **44**, e71; 10.1093/nar/gkv1507 (2016).

37. Santamaría, L. P. & del Peso, L. TFEA. ChIP: Analyze Transcription Factor Enrichment. R package version 1.4.2. https://bioconductor.org/packages/release/bioc/html/TFEA.ChIP.html (2019).

38. Konopka, T. umap: Uniform Manifold Approximation and Projection. R package version 0.2.5.0. https://CRAN.R-project.org/package=umap (2020).

39. Garnier, S. viridis: Default Color Maps from 'matplotlib'. R package version 0.5.1. https://CRAN.R-project.org/package=viridis (2018).

40. Ram, K. & Wickham, H. wesanderson: A Wes Anderson Palette Generator. R package version 0.3.6. https://CRAN.R-project.org/package=wesanderson (2018).
