# Supplementary material for: Mammary cell gene expression atlas links epithelial cell remodeling events to breast carcinogenesis
Source: Commun Biol. 2021 Jun 2;4:660. doi: 10.1038/s42003-021-02201-2 (PMC8172904; doi:10.1038/s42003-021-02201-2)
Supplement: Supplementary file 3 — Description of Supplementary Files [file 42003_2021_2201_MOESM3_ESM.pdf]

Description of Additional Supplementary Files

File name: Supplementary Data 1-11
Description:

Supplementary Data 1: Summary of the experimental conditions of the five scRNAseq datasets of mouse mammary glands.

Supplementary Data 2: Statistics and preprocessing parameters for scRNAseq datasets of mouse and human normal mammary glands obtained in 10X platforms.

Supplementary Data 3: Summary of the preprocessing of scRNAseq datasets of mouse and human normal mammary glands.

Supplementary Data 4: Marker genes for the clusters identified in the mouse integrated data.

Supplementary Data 5: Top gene signatures in mammary lineage differentiations.

Supplementary Data 6: Top correlated genes for the four distinct differentiation status in the mouse mammary epithelium.

Supplementary Data 7: Correlation of the RNA-based features with the four distinct differentiation status in the mouse mammary epithelium.

Supplementary Data 8: Top correlated genes for the three differentiated status in the human breast epithelium.

Supplementary Data 9: Correlation of the RNA-based features with the three differentiated status in the human breast epithelium.

Supplementary data 10: Common and specific lineage genes in mice and humans.

Supplementary Data 11: Results of GSVA analysis for common and species-specific lineage genes.
