# Supplementary material for: Mammary cell gene expression atlas links epithelial cell remodeling events to breast carcinogenesis
Source: Commun Biol. 2021 Jun 2;4:660. doi: 10.1038/s42003-021-02201-2 (PMC8172904; doi:10.1038/s42003-021-02201-2)
Supplement: Supplementary file 5 — Reporting summary [file 42003_2021_2201_MOESM5_ESM.pdf]

nature research

Corresponding author(s): Shiuan Chen

Last updated by author(s): Apr 20, 2021

Reporting Summary

Nature Research wishes to improve the reproducibility of the work that we publish. This form provides structure for consistency and transparency in reporting. For further information on Nature Research policies, see our Editorial Policies and the Editorial Policy Checklist.

Statistics

For all statistical analyses, confirm that the following items are present in the figure legend, table legend, main text, or Methods section.

n/a	Confirmed	
[ ]	[x]	The exact sample size (*n*) for each experimental group/condition, given as a discrete number and unit of measurement
[ ]	[x]	A statement on whether measurements were taken from distinct samples or whether the same sample was measured repeatedly
[ ]	[x]	The statistical test(s) used AND whether they are one- or two-sided<br>*Only common tests should be described solely by name; describe more complex techniques in the Methods section.*
[x]	[ ]	A description of all covariates tested
[ ]	[x]	A description of any assumptions or corrections, such as tests of normality and adjustment for multiple comparisons
[ ]	[x]	A full description of the statistical parameters including central tendency (e.g. means) or other basic estimates (e.g. regression coefficient) AND variation (e.g. standard deviation) or associated estimates of uncertainty (e.g. confidence intervals)
[ ]	[x]	For null hypothesis testing, the test statistic (e.g. *F*, *t*, *r*) with confidence intervals, effect sizes, degrees of freedom and *P* value noted<br>*Give P values as exact values whenever suitable.*
[x]	[ ]	For Bayesian analysis, information on the choice of priors and Markov chain Monte Carlo settings
[x]	[ ]	For hierarchical and complex designs, identification of the appropriate level for tests and full reporting of outcomes
[ ]	[x]	Estimates of effect sizes (e.g. Cohen's *d*, Pearson's *r*), indicating how they were calculated

Our web collection on statistics for biologists contains articles on many of the points above.

Software and code

Policy information about availability of computer code

Data collection	Data collection criteria are stated in the Method section. The complete code and data are published and available at Zenodo (http://doi.org/10.5281/zenodo.4674274).
Data analysis	The following software was used for the purpose: R was run using RStudio Desktop in Windows 10 (ver. 1803) and Python was run using JupyterLab in Ubuntu (ver. 18.04 LTS) built on Windows Subsystems for Linux. The version information and availability of the languages, environments, and packages were summarized in Supplementary Table 1. The complete code and data are published and available at Zenodo (http://doi.org/10.5281/zenodo.4674274).

For manuscripts utilizing custom algorithms or software that are central to the research but not yet described in published literature, software must be made available to editors and reviewers. We strongly encourage code deposition in a community repository (e.g. GitHub). See the Nature Research guidelines for submitting code & software for further information.

Data

Policy information about availability of data

All manuscripts must include a data availability statement. This statement should provide the following information, where applicable:

- Accession codes, unique identifiers, or web links for publicly available datasets
- A list of figures that have associated raw data
- A description of any restrictions on data availability

Data availability

The authors declare that all data supporting the findings of this study are available within the article, the Supplementary Data, and the data repository or from the corresponding author upon reasonable request. The data from the Tabula Muris Consortium was available in the Figshare with the identifier doi.org/10.1038/s41586-018-0590-413,90. The other publicly available scRNA datasets were retrieved from the Gene Expression Omnibus under the following accession codes:

GSE111113 (https://www.ncbi.nlm.nih.gov/geo/query/acc.cgi?acc=GSE111113, Girradi et al.10), GSE103275 (https://www.ncbi.nlm.nih.gov/geo/query/acc.cgi?acc=GSE103275, Pal et al.11), GSE106273 (https://www.ncbi.nlm.nih.gov/geo/query/acc.cgi?acc=GSE106273, Bach et al.12), GSE113197 (https://www.ncbi.nlm.nih.gov/geo/query/acc.cgi?acc=GSE113197, Human normal breast, Nguyen et al.37), and GSE75688 (https://www.ncbi.nlm.nih.gov/geo/query/acc.cgi?acc=GSE75688, human breast cancer, Chung et al.43). The scRNAseq data obtained in this study were deposited in the Gene Expression Omnibus along with their associated meta data (GSE149949, https://www.ncbi.nlm.nih.gov/geo/query/acc.cgi?acc=GSE149949). The integrated data are explorable on the web browser and can be downloaded as Seurat R objects at https://mouse-mammary-epithelium-integrated.cells.ucsc.edu. The Mouse and human FACS-sorted microarray data of the mammary epithelium were also retrieved from the GSE under the code GSE19446 (https://www.ncbi.nlm.nih.gov/geo/query/acc.cgi?acc=GSE19446) and GSE16997 (https://www.ncbi.nlm.nih.gov/geo/query/acc.cgi?acc=GSE16997), respectively7,36. The TCGA breast cancer data was retrieved from the NCI GDC (https://www.cancer.gov/tcga)39. The data and custom codes in this study were deposited and available in Zenodo (http://doi.org/10.5281/zenodo.4674274)69.

Field-specific reporting

Please select the one below that is the best fit for your research. If you are not sure, read the appropriate sections before making your selection.

- [x] Life sciences
- [ ] Behavioural & social sciences
- [ ] Ecological, evolutionary & environmental sciences

For a reference copy of the document with all sections, see nature.com/documents/nr-reporting-summary-flat.pdf

Life sciences study design

All studies must disclose on these points even when the disclosure is negative.

Sample size	The sample size of the study was determined based on the previous study.
Data exclusions	No data was excluded from the study.
Replication	To verify reproducibility of the key data integration process, we input various parameters using different algorithms as described in the Method section. We also compared the results with the data from other species and modalities to verify our findings. Regarding our in vivo experiments, the model was verified by the previous publication and results were supported by the data from others through the integration analysis although the replication of the entire experiment including single-cell RNA seq was not feasible due to its experimental burden.
Randomization	The animals were randomly allocated to the treatment groups.
Blinding	The investigators were blinded to allocate the groups. The evaluation of the results were also performed in a blind fashion.

Reporting for specific materials, systems and methods

We require information from authors about some types of materials, experimental systems and methods used in many studies. Here, indicate whether each material, system or method listed is relevant to your study. If you are not sure if a list item applies to your research, read the appropriate section before selecting a response.

Materials & experimental systems

n/a	Involved in the study	
x		Antibodies
x		Eukaryotic cell lines
x		Palaeontology and archaeology
	x	Animals and other organisms
x		Human research participants
x		Clinical data
x		Dual use research of concern

Methods

n/a	Involved in the study	
x		ChIP-seq
x		Flow cytometry
x		MRI-based neuroimaging

Animals and other organisms

Policy information about studies involving animals; ARRIVE guidelines recommended for reporting animal research

Laboratory animals	Mouse, BALB/cj, female, nine-weeks-old
Wild animals	The study did not involve wild animals.
Field-collected samples	The study did not involve samples collected from the field.
Ethics oversight	The study was approved and overseen by the Institutional Animal Care and Use Committee and performed according to the institutional and NIH guidelines.

Note that full information on the approval of the study protocol must also be provided in the manuscript.
